# Mitophagy Reprograms Lactate Metabolism to Suppress THBS1 via H3K18la Reduction, Alleviating Intervertebral Disc Degeneration

**DOI:** 10.34133/research.0957

**Published:** 2025-11-05

**Authors:** Kanbin Wang, Xiaoyong Wu, Hao Li, Xiaoyan Xu, Feng Cheng, Jinyang Chen, Han Zhou, Jianbin Xu, Chao Yu, Yi Li, Ronghao Wang, Zheng Yuan, Minjun Yao, Xiaoxiao Ji, Ruijie Chen, Xiaopeng Zhou, Bin Han, Chengzhen Liang, Hongguang Xia, Xiaohua Yu, Fangcai Li

**Affiliations:** ^1^ Department of Orthopedic Surgery, The Second Affiliated Hospital, Zhejiang University School of Medicine, Hangzhou, Zhejiang, China.; ^2^ Orthopedics Research Institute of Zhejiang University, Hangzhou, Zhejiang, China.; ^3^ Clinical Research Center of Motor System Disease of Zhejiang Province, Hangzhou, Zhejiang, China.; ^4^ Zhejiang Key Laboratory of Motor System Disease Precision Research and Therapy, Hangzhou City, Zhejiang Province, China.; ^5^ Research Center of Clinical Pharmacy of The First Affiliated Hospital & Liangzhu Laboratory, Zhejiang University School of Medicine, Hangzhou 310003, China.; ^6^ Key Laboratory of Novel Targets and Drug Study for Neural Repair of Zhejiang Province, School of Medicine, Hangzhou City University, Hangzhou, Zhejiang, China.; ^7^ State Key Laboratory of Transvascular Implantation Devices, Hangzhou 310009, China.

## Abstract

Mitophagy alleviates intervertebral disc degeneration (IVDD) by suppressing cGAS–STING and NLRP3 inflammasome-mediated pyroptosis pathways; however, its metabolic regulatory mechanism remains unexplored. Herein, we discovered that mitophagy activator TJ0113 drives metabolic reprogramming characterized by substantially reduced lactate production in senescent nucleus pulposus (NP) cells. This decline directly diminishes histone H3 lysine 18 lactylation (H3K18la), consequently suppressing transcription of the pro-inflammatory gene thrombospondin-1 (THBS1) and blocking downstream inflammatory cascades in IVDD progress. Through combined genetic silencing of THBS1 and pharmacological inhibition of lactate generation, we establish the lactate–H3K18la–THBS1 axis as the essential mechanism mediating mitophagy’s anti-inflammatory effects. Our work provides the first evidence that mitophagy orchestrates a metabolic–epigenetic regulatory axis (lactate–H3K18la–THBS1), unveiling novel therapeutic targets for IVDD and paving the way for epigenetic therapies against disc degeneration.

## Introduction

Low back pain, the most widespread and impactful musculoskeletal ailment, is a leading contributor to worldwide disability [1]. Its occurrence is closely associated with intervertebral disc degeneration (IVDD) [2–4]. During IVDD progression, nucleus pulposus (NP) cells undergo a phenotypic shift from a hydrated, gel-like state to a fibrotic and senescent phenotype, disrupting extracellular matrix (ECM) homeostasis, triggering inflammatory cascades, and accelerating degeneration [5–7]. Recent evidence has highlighted mitochondrial damage as a critical factor contributing to NP cell senescence and IVDD progression [8,9]. Impaired mitochondrial function promotes the release of mitochondrial DNA (mtDNA) and excessive production of reactive oxygen species (ROS), which activate the cGAS–STING and NLRP3 inflammasome-dependent pyroptosis pathways, thereby promoting inflammatory signaling, NP cell senescence, and IVDD progression [6,10–12]. Thus, maintaining mitochondrial homeostasis in NP cells may represent a promising therapeutic strategy for the treatment of IVDD.

Mitophagy—the selective autophagic clearance of damaged mitochondria—has emerged as a critical protective mechanism in IVDD [13,14]. As a specialized form of autophagy, mitophagy is primarily regulated by the canonical PINK–PARKIN pathway and the receptor-mediated noncanonical pathway (such as BNIP3, BNIP3L/NIX, BCL2L13, FUNDC1, and MCL-1), which are activated in response to mitochondrial depolarization or hypoxic stress. Through these pathways, damaged mitochondria are selectively labeled and targeted for lysosomal degradation, maintaining mitochondrial integrity and cellular homeostasis [15,16]. By removing damaged mitochondria, mitophagy attenuates ROS accumulation, restores redox balance, and preserves oxidative phosphorylation to support the high energy demand of NP cells [17]. In addition, mitophagy limits the release of mitochondrial mtDNA, thereby suppressing cGAS–STING and NLRP3 inflammasome activation, which are major triggers of chronic inflammation and pyroptosis in IVD [6,11,18]. Moreover, efficient mitochondrial turnover helps prevent apoptosis and senescence-associated secretory phenotype (SASP) development, sustains ECM anabolism, and maintains the regenerative potential of NP cells [9]. However, the metabolic regulatory mechanisms through which mitophagy exerts these anti-IVDD effects remain unexplored.

Cellular metabolic reprogramming has emerged as a central regulator of cell fate and inflammatory responses, particularly under conditions of senescence and disease [19–22]. A compensatory shift toward glycolysis is commonly observed in senescent NP cells, which is primarily attributed to damaged mitochondria. This metabolic shift results in the accumulation of glycolytic metabolite lactate [23,24]. The perception of lactate has shifted from that of a metabolic end-product to a key regulator of various cellular activities. Recent findings have uncovered histone lactylation—a novel lactate-driven epigenetic mechanism, which directly regulates gene transcription [25]. Notably, the association between histone lactylation and the transcriptional activation of inflammatory genes has garnered growing attention [26,27]. This lactate-driven metabolic–epigenetic regulatory axis provides a mechanistic bridge between metabolic changes and inflammatory gene expression during aging and disease progression.

Given the emerging role of metabolic reprogramming and lactate signaling in inflammation-associated IVDD, we hypothesized that mitophagy might mitigate IVDD progress by modulating a lactate–histone lactylation axis to control key pro-inflammatory gene expression in senescent NP cells. In this study, we aimed to explore whether mitophagy reprograms metabolic profile in senescent NP cells, with a particular focus on its regulation of lactate production. Our results identify a previously unrecognized metabolic–epigenetic axis (lactate–H3K18la–THBS1) as the essential mechanism mediating mitophagy’s suppression of inflammatory cascades in IVDD. This work establishes a theoretical framework for targeting the metabolic–epigenetic network in disc degeneration therapy.

## Results

### IVDD is associated with mitochondrial dysfunction

**Fig. 1.**
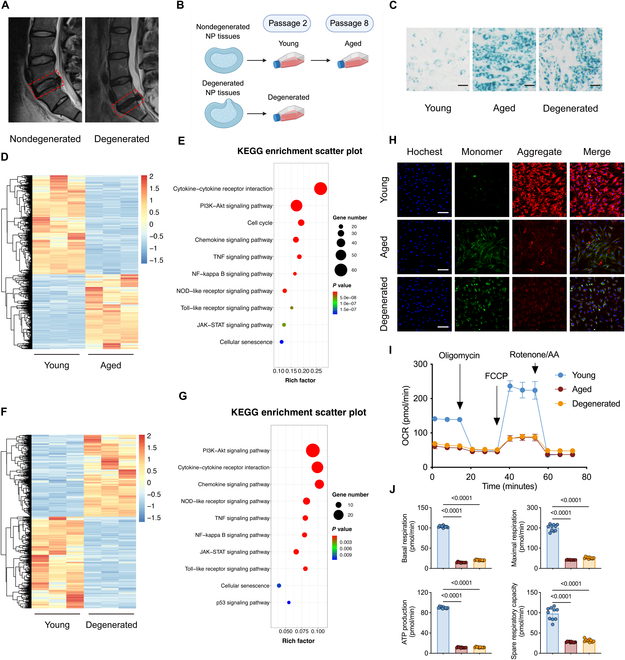
Intervertebral disc degeneration (IVDD) is associated with mitochondrial dysfunction. (A) Representative T2-weighted magnetic resonance imaging (MRI) images were assessed using the Pfirrmann grading system. Grade I or II was classified as the nondegenerated group. Grade IV was classified as the degenerated group. (B) Schematic of cell extraction and culture. (C) Representative SA-β-gal staining images of NP cells. Scale bar, 20 μm. (D) Hierarchical clustering revealed prominent separation between young and aged samples. (E) KEGG pathway enrichment analysis of RNA-seq data (*n* = 3) was conducted to compare inflammation- and senescence-related differentially expressed genes (DEGs) between young and aged NP cells. (F) Hierarchical clustering analysis revealed major differences between young and degenerated groups. (G) KEGG enrichment analysis of RNA sequencing (*n* = 3) was conducted to compare inflammation- and senescence-related DEGs between young and degenerated groups. (H) Representative images of JC-1 staining. Scale bar, 50 μm. (I and J) Seahorse metabolic analysis (OCR) of NP cells of different groups. Results represent the mean ± SD of at least 3 independent experiments. Significance levels are shown within the graphs.

The Pfirrmann grading system revealed a progressive decrease in T2-weighted high-intensity white signals, which became heterogeneous and were eventually replaced by high-intensity black signals. This radiographic progression reflects decreased water content and increased fibrosis in NP tissue during IVDD. NP tissue samples were obtained from clinical spinal surgeries and classified according to Pfirrmann grades: grades I and II (nondegenerated group) and grade IV (degenerated group) (Fig. 1A). Primary NP cells isolated from the nondegenerated group at passage 2 (P2) served as young controls (young NP cells). Serial passaging to P8 generated an in vitro aged model (aged NP cells), while P2 cells from degenerated group specimens represented naturally degenerated cells (degenerated NP cells) (Fig. 1B). Senescence-associated β-galactosidase (SA-β-gal) staining intensity was markedly enhanced in both aged and degenerated cells compared to young controls (Fig. 1C and Fig. S1A). Kyoto Encyclopedia of Genes and Genomes (KEGG) enrichment analysis of RNA sequencing (RNA-seq) demonstrated marked enrichment of inflammation- and senescence-related differentially expressed genes (DEGs) in aged and degenerated groups (Fig. 1D to G). We further explored the correlation between the DEGs from 2 RNA-seq data by performing an intersection analysis. We identified 450 overlapping DEGs shared between the 2 comparisons. To gain insight into the potential biological relevance of these overlapping genes, we conducted KEGG pathway enrichment analysis. Notably, among the top 30 enriched pathways, we observed a marked enrichment of multiple inflammation-related signaling pathways, which further supports the link between cellular senescence and IVDD (Fig. S1C and D).

Existing research suggests that mitochondrial dysfunction plays a central role in NP cell senescence and IVDD progression [5,9]. Consequently, we systematically evaluated mitochondrial function in NP cells. JC-1 probe analysis showed markedly reduced red fluorescence intensity coupled with increased green fluorescence in aged and degenerated cells compared to young controls, indicating mitochondrial membrane potential depolarization (Fig. 1H and Fig. S1B). Seahorse metabolic analysis [oxygen consumption rate (OCR)] confirmed impaired mitochondrial function in both aged and degenerated NP cells versus young controls, as evidenced by the decreased basal respiration, maximal respiration, adenosine triphosphate (ATP) production, and spare respiratory capacity (Fig. 1I and J). These results collectively demonstrate the accumulation of dysfunctional mitochondria during NP cell senescence and IVDD progression.

### Pharmacological activation of mitophagy delays IVDD in rat models

**Fig. 2.**
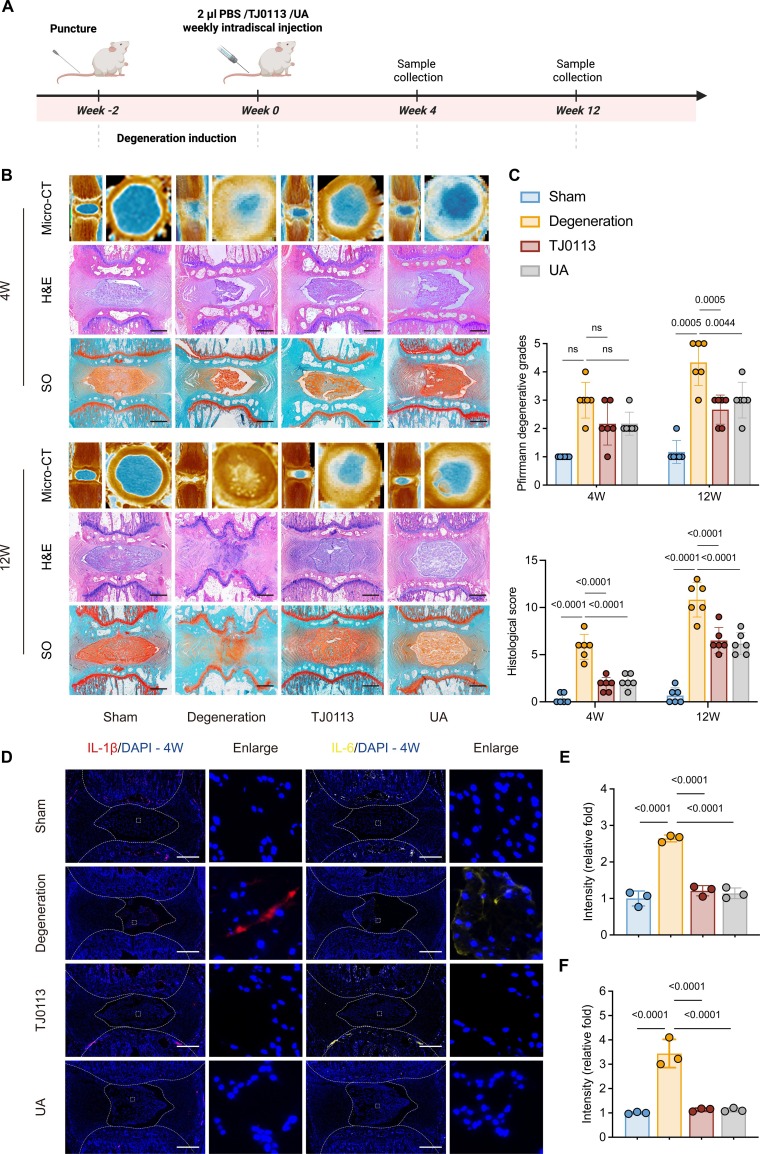
Pharmacological activation of mitophagy delays IVDD in rat models. (A) Schematic illustration of the experimental design. (B) Representative images of micro-CT, H&E staining, and SO&FG staining of rat coccygeal IVDs. Scale bar, 1 mm. (C) Pfirrmann degenerative grades (*n* = 6) and histological score (*n* = 6) of rat IVDs. (D) Representative immunofluorescence (IF) images of SASP factors in rat coccygeal IVDs at 4 weeks after treatment. Scale bar, 500 μm. (E and F) Quantification of interleukin-1β (IL-1β) and IL-6 in rat coccygeal IVDs at 4 weeks after treatment. Results represent the mean ± SD of at least 3 independent experiments. Significance levels are shown within the graphs.

Given the accumulation of damaged mitochondria in senescent NP cells, pharmacological activation of mitophagy represents a promising approach to restore mitochondrial homeostasis and mitigate senescence-associated phenotypes. To evaluate the therapeutic effect of mitophagy activation on IVDD, we employed 2 well-established mitophagy activators—urolithin A (UA) [28,29] and TJ0113 [30,31] (a modified compound derived from UMI-77 [15])—to induce mitophagy in vivo. In our preliminary experiments, both TJ0113 (10 and 20 mg/ml) and UA (5 and 7.5 mg/ml) effectively alleviated IVDD progression. To ensure safety and minimize potential adverse effects, we selected the lower concentrations of TJ0113 and UA for the subsequent in vivo studies (Fig. S2A and B). The experimental timeline and dosing schedule were presented in Fig. 2A. We assessed mitophagy by evaluating Tom20 and Lamp1 colocalization in IVD tissues, which confirmed that both UA and TJ0113 enhanced mitophagy in vivo (Fig. S2C). X-ray and micro-computed tomography (CT) analysis showed that mitophagy activation substantially restored the height of rat coccygeal IVDs and maintained better-preserved NP morphology compared to the degeneration group (Fig. 2B and C and Fig. S2D and E). Histological degeneration was assessed using hematoxylin and eosin (H&E) and safranin O and fast green (SO&FG) staining. Compared to the degenerated group, TJ0113-treated and UA-treated groups exhibited better preservation of NP morphology, ECM integrity, and a clearer boundary between the NP and annulus fibrosus (AF) (Fig. 2B and C). Moreover, immunofluorescence (IF) analysis revealed that SASP-related inflammatory cytokines were substantially reduced in the TJ0113-treated and UA-treated groups compared to the degeneration group (Fig. 2D to F). These findings indicate that pharmacological activation of mitophagy in vivo markedly down-regulates SASP factors and delays IVDD progression.

### Mitophagy activation reduces SASP factors and improves mitochondrial quality in senescent NP cells

**Fig. 3.**
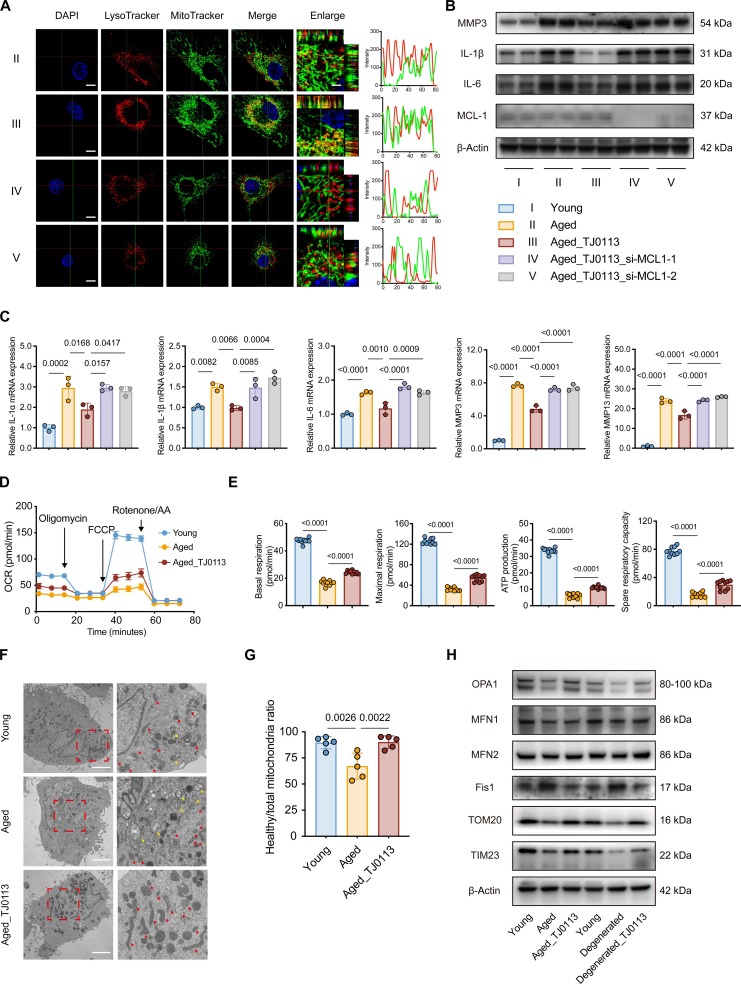
Mitophagy activation reduces SASP factors and improves mitochondrial quality in senescent NP cells. (A) IF analysis and quantification of MitoTracker and LysoTracker in aged NP cells treated with or without TJ0113 (5 μM) after knocking down MCL1. Scale bars, 10 μm. (B) Western blot analysis of SASP factors (IL-1β, IL-6, and MMP3) and MCL1 in young and aged NP cells treated with TJ0113 or not (5 μM, 48 h) after knocking down MCL1. (C) Quantitative reverse transcription PCR (qRT-PCR) analysis of SASP factors in young and aged NP cells treated with TJ0113 or not (5 μM, 48 h; *n* = 3) after knocking down MCL1. (D and E) Seahorse metabolic analysis (OCR) of aged NP cells treated with TJ0113 or not (5 μM, 48 h). Young group served as control. (F and G) Representative transmission electron microscopy (TEM) images and quantitative analysis of healthy mitochondria numbers (5 μM). Young NP cells served as control. Scale bar, 5 μm. (H) Western blot analysis of mitochondrial dynamic markers and mitochondrial membrane proteins in aged and degenerated NP cells treated with TJ0113 or not (5 μM, 48 h). Results represent the mean ± SD of at least 3 independent experiments. Significance levels are shown within the graphs.

Next, we induced mitophagy in vitro using TJ0113 and UA to investigate the therapeutic effect on senescent NP cells. Notably, TJ0113 [30,31], a long-acting mitophagy activator derived from the structural optimization of UMI-77 [15], promotes mitophagy by targeting the mitophagy receptor MCL1. To exclude the potential involvement of apoptosis in the effects of TJ0113 on senescent NP cells, flow cytometry was employed to identify the optimal concentration for inducing mitophagy. The results supported using 5 μM TJ0113 in subsequent experiments (Fig. S3). Using fluorescent labeling of mitochondria and lysosomes, we monitored the mitophagy process. TJ0113 increased the colocalization of mitochondrial and lysosomal fluorescence signals, indicating that TJ0113 effectively induces mitophagy in senescent NP cells. However, this effect was suppressed upon MCL1 knockdown (Fig. 3A and Fig. S4A). Moreover, TJ0113 treatment markedly reduced inflammatory factors and matrix-degrading enzymes (MMP3 and MMP13) in senescent NP cells. Notably, the inhibitory effect of TJ0113 on SASP factors was markedly attenuated upon MCL1 perturbation (Fig. 3B and C and Fig. S4B and C). Moreover, UA was a well-recognized mitophagy activator that induced mitophagy via the Pink1/Parkin pathway. Similarly, UA treatment markedly reduced SASP factors in senescent NP cells. Notably, the inhibitory effect of UA on SASP factors was attenuated following Pink1 knockdown (Fig. S5A and B). In summary, these results indicate that pharmacological activation of mitophagy, by either TJ0113 or UA, markedly reduces SASP factors in senescent NP cells.

Considering the recent identification of TJ0113 as a potent mitophagy activator with efficacy comparable to UA, we chose TJ0113 for the activation of mitophagy in our subsequent mechanistic explorations. TJ0113 treatment increases basal and maximal respiration levels, ATP production, and spare respiratory capacity in aged and degenerated NP cells (Fig. 3D and E and Fig. S6A and B). JC-1 staining showed that TJ0113 treatment in aged and degenerated cells restored mitochondrial membrane potential (Fig. S6C). Transmission electron microscopy (TEM) showed that aged NP cells exhibited marked smaller mitochondria with widespread swelling, indicative of mitochondrial fragmentation and dysfunction. In contrast, TJ0113 treatment restored normal mitochondrial morphology and markedly enhanced mitochondrial fusion (Fig. 3F and G). The relative mtDNA copy number in NP cells increased with treatment of TJ0113, indicating enhanced mitochondrial biogenesis (Fig. S6D). Mitochondrial fusion protein OPA1 and mitochondrial components (TOM20 and TIM23) were up-regulated, while mitochondrial fission protein Fis1 was down-regulated following TJ0113 treatment. Interestingly, MFN1 and MFN2 remained unchanged (Fig. 3H). Collectively, these findings suggest that TJ0113 improves cellular phenotypes associated with aging in senescent NP cells through the regulation of mitochondrial homeostasis.

### Metabolic reprogramming represents a key response to mitophagy activation

**Fig. 4.**
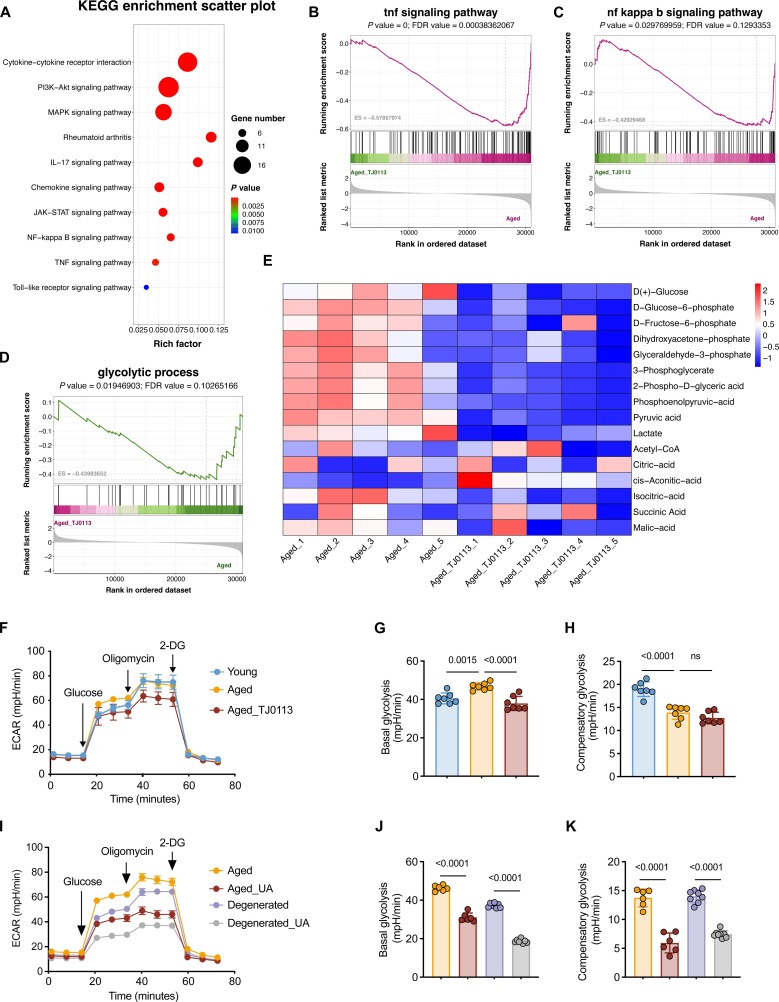
Metabolic reprogramming represents a key response to mitophagy activation. (A) RNA-seq analysis of aged NP cells treated with or without TJ0113 (5 μM, 48 h; *n* = 3). KEGG pathway enrichment analysis of RNA-seq data (*n* = 3) was conducted to compare inflammation-related DEGs. (B and C) GSEA showed significant enrichment of the “TNF signaling pathway” and “NF-κB signaling pathway” in TJ0113-treated aged NP cells. (D) GSEA showed significant enrichment of the “glycolytic process” in TJ0113-treated aged NP cells versus controls. (E) Heatmap of LC-MS data showing metabolite changes in glycolysis and tricarboxylic acid (TCA) cycle between TJ0113-treated and untreated aged NP cells (*n* = 5). Glycolytic metabolites (blue) were markedly reduced in TJ0113-treated aged NP cells versus controls. (F to H) Seahorse metabolic analysis (ECAR) of aged NP cells treated with TJ0113 or not (5 μM, 48 h). Young NP cells served as control. (I to K) Seahorse metabolic analysis (ECAR) of aged and degenerated NP cells treated with or without urolithin A (UA; 10 μM, 48 h). Results represent the mean ± SD of at least 3 independent experiments. Significance levels are shown within the graphs.

Although suppression of the cGAS–STING pathway and pyroptosis is a well-established mechanism by which mitophagy alleviates inflammatory degeneration in NP cells, accumulating evidence suggests that this pathway does not fully account for the broad cellular effects of mitophagy. In particular, whether metabolic changes mediate the protective role of mitophagy remains largely unexplored and is the central focus of this study. We performed RNA-seq analysis on TJ0113-treated and untreated aged NP cells. A total of 345 transcripts were significantly up-regulated (2-fold, *P* < 0.05), and 563 transcripts were significantly down-regulated (2-fold, *P* < 0.05) in the Aged_TJ0113 group (Fig. S7A). KEGG pathway analysis revealed notable enrichment in inflammation-related pathways (Fig. 4A). Gene set enrichment analysis (GSEA) further demonstrated that TJ0113 treatment down-regulated key pro-inflammatory signaling pathways, including the tumor necrosis factor (TNF) and nuclear factor-κB (NF-κB) signaling pathways (Fig. 4B and C), corroborating its role in suppressing inflammatory responses in senescent NP cells. In addition, given the marked enhancement of mitochondrial function by TJ0113 treatment, we conducted metabolomic profiling in senescent NP cells following the same treatment protocol and performed integrated analysis with RNA-seq data. GSEA demonstrated significant enrichment of the “glycolytic process” in aged NP cells compared to young controls (Fig. S7B). Consistent with prior reports, NP cells undergoing senescence are characterized by up-regulated glycolysis and down-regulated oxidative phosphorylation [23]. Our data demonstrated that activation of mitophagy effectively reversed this metabolic pattern shift and induced a metabolic profile reminiscent of “younger” state in senescent NP cells. GSEA showed significant down-regulation of the “glycolytic process” pathway in TJ0113-treated aged NP cells (Fig. 4D). Corroborating this, targeted metabolomics analysis showed marked reductions in key glycolytic metabolites in the TJ0113-treated aged NP cells versus controls (Fig. 4E), further confirming the attenuation of glycolytic flux. We further assessed the glycolytic function in NP cells and observed that basal glycolysis was significantly up-regulated in aged and degenerated NP cells compared to young controls. Notably, TJ0113 treatment not only reversed this hyperglycolytic state but also reduced basal glycolysis to levels below those of young controls (Fig. 4F to H and Fig. S7C to E). In addition, we treated senescent NP cells with UA. Consistent with the effect of TJ0113, UA treatment significantly reduced both basal and compensatory glycolysis in aged and degenerated NP cells (Fig. 4I to K). These results further support our conclusion that mitophagy activation drives metabolic reprogramming in senescent NP cells, characterized by attenuated glycolysis and up-regulated mitochondrial function.

### Down-regulation of lactate production and histone lactylation is driven by mitophagy activation

**Fig. 5.**
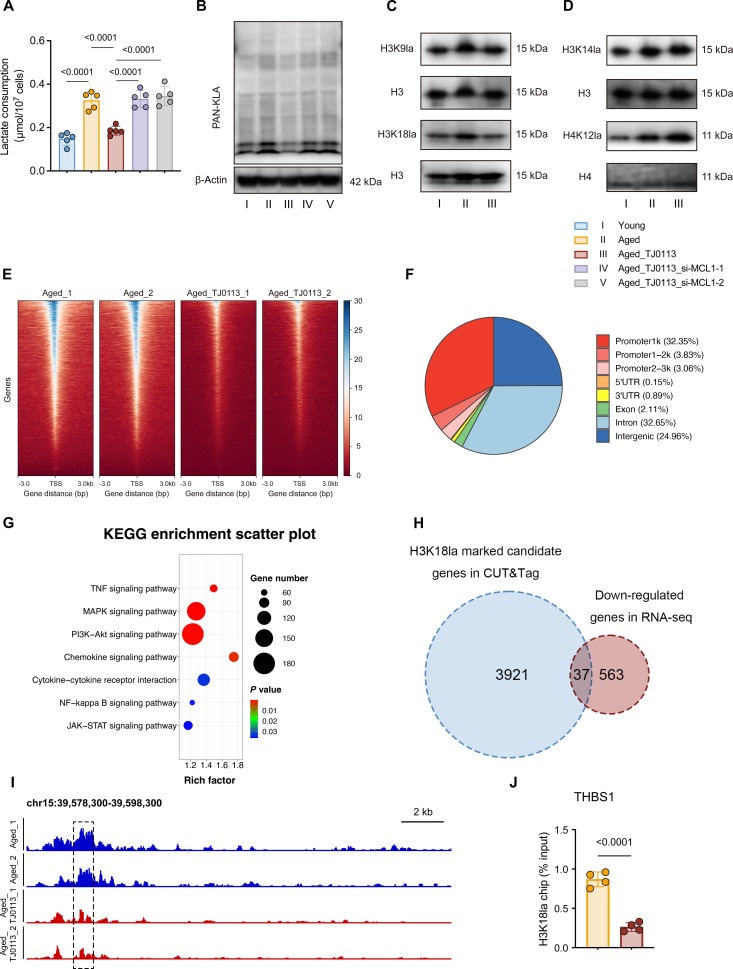
Down-regulation of lactate production and histone lactylation is driven by mitophagy activation. (A) Lactate levels of the young and aged NP cells treated with or without TJ0113 (5 μM, 48 h; *n* = 5) after knocking down MCL1. (B) Western blot analysis of PAN-KLA in young and aged NP cells treated with or without TJ0113 (5 μM, 48 h) after knocking down MCL1. (C and D) Western blot analysis of lysine lactylation markers (H3K9la, H3K14la, H3K18la, H4K12la) treated with TJ0113 or not (5 μM, 48 h). Histone H3/H4 served as loading controls. (E) Heatmap depicting H3K18la genomic occupancy profiles [±3 kb flanking transcription start sites (TSSs)] treated with TJ0113 or not (5 μM, 48 h). Genes are ranked vertically by descending H3K18la signal intensity. (F) Comparative pie charts showing genome-wide differential H3K18la distribution patterns at annotated genomic regions in aged NP cells treated with TJ0113 (5 μM, 48 h) compared to untreated controls. (G) KEGG enrichment analysis of H3K18la-marked genes. (H) Venn diagram depicting genes down-regulated in RNA-seq analysis of aged NP cells treated with TJ0113 (5 μM, 48 h) compared to untreated controls and the down-regulated target genes bound by H3K18la under identical treatment conditions. (I) Normalized read densities for H3K18la at the THBS1 genes. (J) ChIP-qPCR assay in aged NP cells. Results represent the mean ± SD of at least 3 independent experiments. Significance levels are shown within the graphs.

Given that mitophagy activation drives metabolic reprogramming and reduces SASP factors in senescent NP cells, we further investigated the mechanistic link between metabolic alterations and epigenetics. Among the key glycolytic metabolites altered by TJ0113 treatment, we focused specifically on lactate due to its role as a critical mediator of metabolism–epigenetics crosstalk [25,26,32]. Therefore, we measured intracellular lactate levels across experimental groups. Consistent with previous reports, aged NP cells exhibited significantly higher lactate levels compared to young controls. TJ0113 treatment effectively reduced lactate accumulation in aged NP cells relative to untreated controls. Notably, the inhibitory effect of TJ0113 on lactate level was attenuated upon MCL1 perturbation (Fig. 5A). Given lactate’s established role in driving histone lactylation [25], we further examined this epigenetic modification. Aged NP cells demonstrated elevated histone lactylation levels compared to young controls, while TJ0113 treatment markedly attenuated this increase. Notably, the reduction in histone lactylation induced by TJ0113 was impaired upon MCL1 knockdown (Fig. 5B). Similarly, UA treatment markedly reduced both intracellular lactate levels and histone lactylation in aged NP cells. These effects were reversed upon Pink1 knockdown (Fig. S8A and B). We then examined multiple canonical histone lactylation sites (H3K9la, H3K14la, H3K18la, and H4K12la) and found that H3K18la showed the most pronounced alteration following TJ0113 treatment (Fig. 5C and D). This marked response indicates that H3K18la may serve as a key mediator of mitophagy’s effects in aged NP cells. To identify potential downstream genes regulated through H3K18la lactylation, we performed cleavage under targets and tagmentation (CUT&Tag) sequencing. Genomic localization analysis showed that H3K18la modifications were predominantly enriched in promoter and upstream regulatory regions (Fig. 5E). In TJ0113-treated aged NP cells, we identified 3,921 genes with decreased H3K18la occupancy and 662 genes with increased H3K18la modification (Fig. S8C). In total, 39.24% of these differentially modified sites were localized to promoter regions (Fig. 5F). Pathway enrichment analysis via KEGG showed a strong association between H3K18la-modified genes and inflammatory signaling pathways (Fig. 5G). Integrated analysis of CUT&Tag and RNA-seq data identified 37 down-regulated genes that showed concurrent reduction in both H3K18la modification and transcriptional expression (Fig. 5H). Among these 37 candidate genes, only 10 genes exhibited H3K18la binding at their promoter regions. According to *Q*-value ranking from RNA-seq analysis, THBS1 was identified as the top candidate (Fig. S8D). This selection was further supported by existing literature demonstrating THBS1’s role in mediating inflammatory responses [33–35] and its established association with IVDD [36]. Collectively, we identified THBS1 as a high-priority target gene potentially regulated through H3K18la (Fig. 5I). Chromatin immunoprecipitation (ChIP)–quantitative polymerase chain reaction (qPCR) confirmed the regulation of THBS1 expression by H3K18la (Fig. 5J). Taken together, these data reveal that mitophagy activation reduces lactate levels and improves cellular senescent phenotypes via the regulation of histone lactylation.

### A mitophagy-driven metabolic–epigenetic axis suppresses SASP factors

**Fig. 6.**
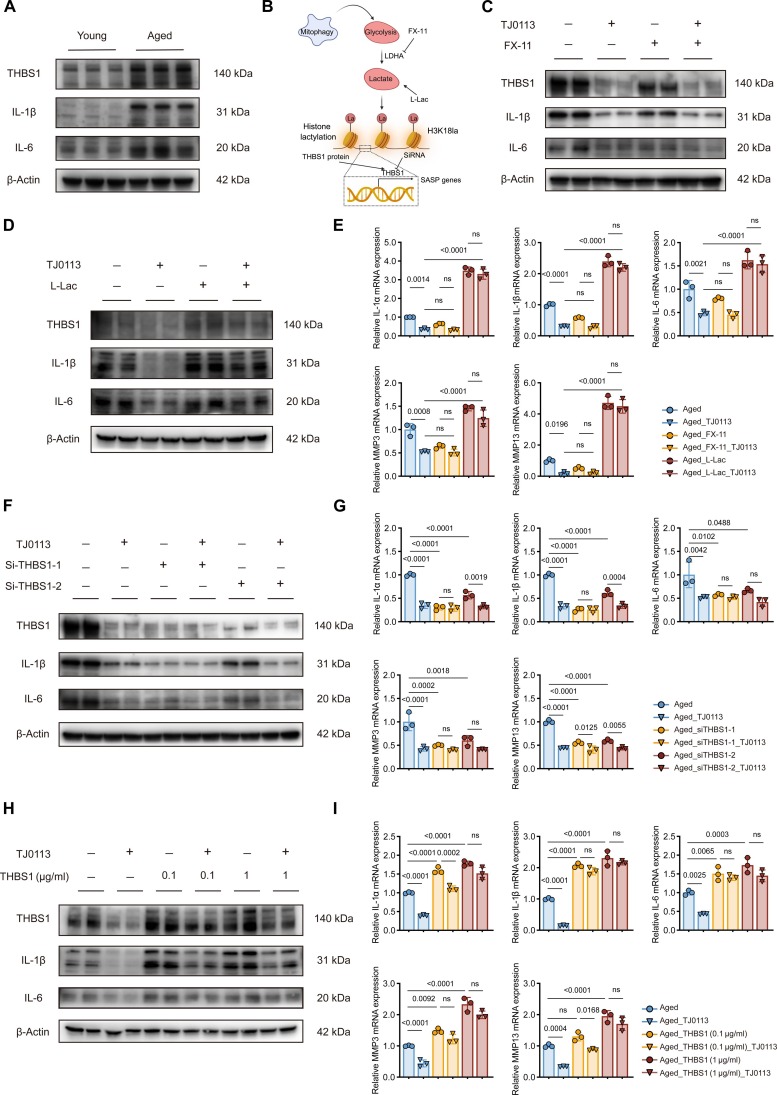
The mitophagy-driven metabolic–epigenetic axis suppresses SASP factors in senescent NP cells. (A) Western blot analysis of SASP factors and thrombospondin-1 (THBS1) in young and aged NP cells. (B) Schematic illustration of the experimental design. (C) Western blot analysis of SASP factors and THBS1 in aged NP cells treated with vehicle, TJ0113 (5 μM), FX-11(20 μM), or both. (D) Western blot analysis of SASP factors and THBS1 in aged NP cells treated with vehicle, TJ0113 (5 μM), L-Lac (10 μM), or both. (E) qRT-PCR analysis of SASP factors in aged NP cells with treatment as (B) and (C) (*n* = 3). (F) Western blot analysis of SASP factors and THBS1 in control and THBS1 knockdown aged NP cells treated with or without TJ0113 (5 μM, 48 h). (G) qRT-PCR analysis of SASP factors in control and THBS1 knockdown aged NP cells treated with TJ0113 or not (5 μM, 48 h) (*n* = 3). (H) Western blot analysis of SASP factors and THBS1 in control and THBS1-treated (0.1/1 μg/ml, 48 h) aged NP cells under treatment with or without TJ0113 (5 μM, 48 h). (I) qRT-PCR analysis of SASP factors in control and THBS1-treated (0.1/1 μg/ml, 48 h) aged NP cells under treatment with or without TJ0113 (5 μM, 48 h) (*n* = 3). Results represent the mean ± SD of at least 3 independent experiments. Significance levels are shown within the graphs.

During NP cell senescence, up-regulated THBS1 expression was accompanied by increased SASP production (Fig. 6A). To determine whether the lactate–H3K18la–THBS1 metabolic–epigenetic axis mediates the mitophagy-induced reduction of SASP factors in senescent NP cells, we conducted both knockout/inhibit and rescue experiments targeting these 2 key nodes: lactate and THBS1 expression (Fig. 6B). We treated the NP cells with FX-11, a specific inhibitor of lactate dehydrogenase A (LDHA), to reduce lactate production and suppress histone lactylation. FX-11 treatment reduced the mRNA and protein expression of THBS1 and SASP factors compared to untreated controls, which was similar to the effect of TJ0113 treatment. Notably, the combination of FX-11 and TJ0113 showed no marked difference in effect compared to TJ0113 treatment alone, suggesting that they operate through overlapping pathways (Fig. 6C and E and Fig. S9A). Conversely, exogenous lactate supplementation in aged NP cells completely reversed TJ0113-mediated suppression, markedly up-regulating both mRNA and protein expression of THBS1 and SASP factors (Fig. 6D and E and Fig. S9A). THBS1 knockdown via small interfering RNA (siRNA) markedly reduced the mRNA and protein expression levels of SASP factors in aged NP cells, which was similar to the TJ0113 treatment (Fig. 6F and G and Fig. S9B). Conversely, exogenous THBS1 recombinant protein supplementation markedly reversed TJ0113-mediated SASP suppression (Fig. 6H and I). Therefore, our findings suggest that the mitophagy-driven metabolic–epigenetic cascade plays a crucial role in decreasing SASP factors in senescent NP cells.

### Targeting the lactate–H3K18la–THBS1 axis attenuates IVDD

**Fig. 7.**
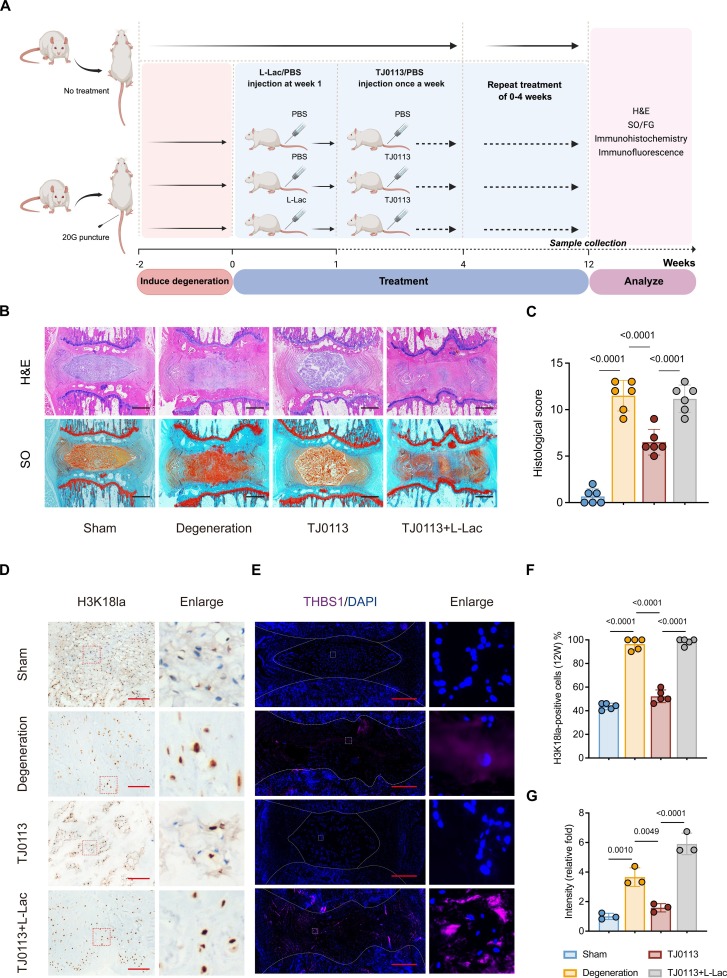
Targeting the lactate–H3K18la–THBS1 axis attenuates IVDD. (A) Schematic illustration of the experimental design. (B) Representative images of H&E and SO&FG staining of rat coccygeal IVDs at 12 weeks after treatment. Scale bar, 1 mm. (C) histological score (*n* = 6) of rat coccygeal IVDs. (D) Representative immunohistochemistry images of H3K18la in rat coccygeal IVDs at 12 weeks after treatment. Scale bar, 200 μm. (E) Representative IF images of THBS1 in rat coccygeal IVDs at 12 weeks after treatment. Scale bar, 500 μm. (F and G) Quantification of SASP factors in rat coccygeal IVDs. Results represent the mean ± SD of at least 3 independent experiments. Significance levels are shown within the graphs.

**Fig. 8.**
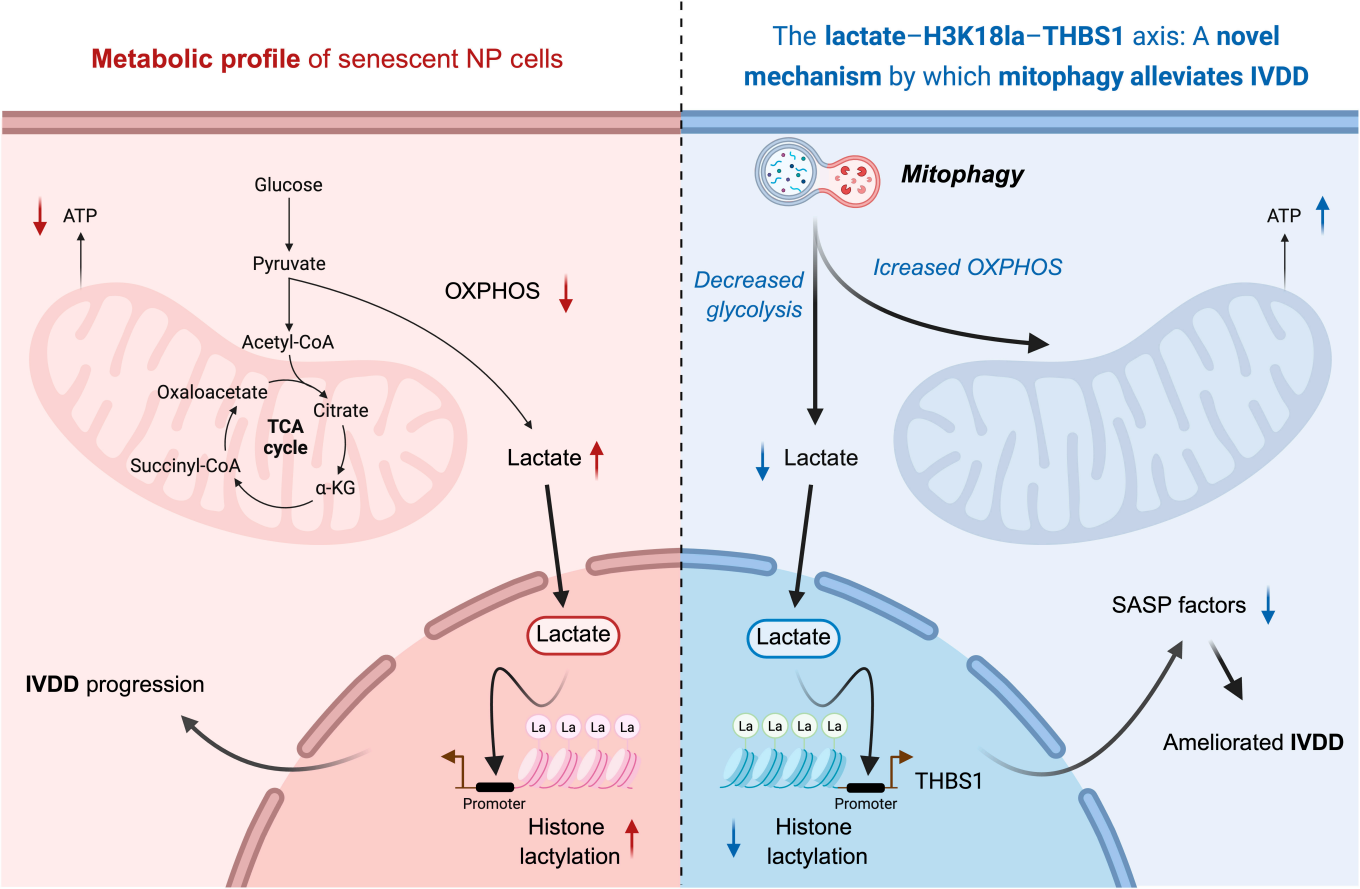
Molecular mechanism of mitophagy activation in alleviating IVDD via metabolic–epigenetic cascade.

To further explore whether the lactate–H3K18la–THBS1 axis mediates the in vivo effect of pharmacological mitophagy activation in delaying IVDD, we administered lactate supplementation in conjunction with TJ0113 treatment. The detailed experimental design is shown in Fig. 7A. Histological degeneration was assessed by using H&E and SO&FG staining. The TJ0113-treated group showed better preservation of NP morphology, ECM integrity, and a clearer boundary between the NP and AF compared to the degenerated group. However, the therapeutic effect was significantly impaired when TJ0113 was co-administered with L-lactate (L-Lac) (Fig. 7B and C). Immunohistochemical (IHC) and IF analysis indicated that TJ0113 treatment alone significantly reduced H3K18la-positive cell rate and down-regulated THBS1 expression compared to the degeneration group. Critically, these effects were reversed when TJ0113 was co-administered with L-Lac, suggesting that exogenous L-Lac restored H3K18la modification and downstream THBS1 protein levels (Fig. 7D to G). These data confirm that mitophagy activation markedly attenuates IVDD via the lactate–H3K18la–THBS1 axis in vivo (Fig. 8).

## Discussion

Here, our findings highlight an unexpected role for mitophagy in the metabolic regulation of NP cell inflammatory aging. Traditionally, mitophagy is regarded as a key process that alleviates NP cell senescence during IVDD procession by improving mitochondrial quality. In our study, we find that a metabolic–epigenetic cascade serves as a mediator for mitophagy in regulating IVDD progression. Senescent NP cells predominantly contain fragmented mitochondria and exhibit a metabolic shift toward glycolysis for energy production [23]. TJ0113 activates mitophagy and triggers metabolic–epigenetic reprogramming, which subsequently counteracts inflammatory senescence in NP cells. Our findings not only reveal mitophagy as a central metabolic switch in senescent NP cell reprogramming but also establish a novel therapeutic strategy targeting the “mitophagy-metabolic–epigenetic axis”, providing a theoretical foundation for IVDD drug development.

Mitophagy, a selective form of autophagy, maintains mitochondrial homeostasis by specifically targeting and degrading damaged or depolarized mitochondria [37]. Extensive preclinical and clinical studies have demonstrated the therapeutic potential of mitophagy activators in neurodegenerative diseases, metabolic syndromes, and cardiovascular disorders [15,16,28,29,38,39]. In the context of IVDD, both genetic and pharmacological induction of mitophagy are regarded as promising therapeutic strategies to clear dysfunctional mitochondria and restore energy metabolism [5,40]. However, recent evidence highlights the “double-edged sword” nature of mitophagy: Excessive activation induced by prolonged mechanical stress or oxidizing stressors can lead to mitochondrial over-clearance, accelerating NP cell senescence and apoptosis [41,42]. Notably, these paradoxical outcomes share a common mechanism—mitophagy activation is invariably triggered by preexisting mitochondrial damage. Therefore, we should highlight a critical consideration for therapeutic strategy design that mitophagy should be activated without inflicting additional mitochondrial injury. To address this limitation, we previously developed UMI-77 [15], a small-molecule compound that selectively activates mitophagy without inducing mitochondrial damage. Through structural optimization of UMI-77, we engineered TJ0113 [30,31], which is currently in phase 2 clinical trials (NCT06596005) and has demonstrated a markedly prolonged in vivo half-life, making it an ideal candidate for long-term therapeutic evaluation in animal models.

Senescence of NP cells is accompanied by mitochondrial dysfunction and the leakage of mtDNA, which activate the cGAS–STING pathway and NLRP3 inflammasome-dependent pyroptosis, thereby triggering inflammatory responses that ultimately drive IVDD. Emerging evidence has shown that mitophagy was essential for the selective clearance of dysfunctional mitochondria and the prevention of mtDNA leakage, thus mitigating the activation of these pro-inflammatory pathways and alleviating IVDD progression [6,11,18,43]. However, in our study, we focused more on how mitophagy alleviates IVDD through metabolic regulation rather than its canonical role in inflammation control. There is evidence indicating that down-regulation of Numb suppresses Parkin-mediated mitophagy, leading to metabolic reprogramming and increased lactate production, which subsequently enhances histone lactylation and alters cancer cell fate [44]. Building upon the evidence, we systematically investigated the impact of mitophagy activation on metabolic reprogramming in senescent NP cells. Integrated transcriptomic and metabolomic analyses demonstrated that mitophagy activation reshaped the metabolic profile of senescent NP cells, characterized by enhanced mitochondrial function and suppressed glycolytic activity. Consistently, extracellular acidification rate (ECAR) assays further confirmed that TJ0113-treated cells exhibited reduced basal glycolytic activity and lactate levels compared with untreated controls. To exclude potential off-target effects of TJ0113, we performed parallel experiments using UA, a well-established selective inducer of mitophagy. Notably, UA treatment similarly suppressed glycolytic activity and lactate levels in NP cells, with trends paralleling those observed with TJ0113. Collectively, this multi-dimensional evidence conclusively establishes that mitophagy activation serves as the central mechanism driving metabolic reprogramming in senescent NP cells.

Aberrant lactate accumulation within the IVD is considered a hallmark of IVDD [24]. Lactate, formerly regarded as a metabolic waste, has been identified as a modulator of histone lactylation, revealing a novel epigenetic mechanism that influences gene expression and plays a role in cancer progression, immune regulation, and chronic inflammatory responses [25,26,44]. In this study, we find that mitophagy is a key metabolic–epigenetic modulator, with H3K18la serving as a major histone lactylation site mediating this process. Although we observed that major histone lactylation sites such as H3K9la, H3K14la, and H4K12la remained largely unchanged upon mitophagy activation, other histone lactylation sites may still contribute to this process. The H3K18la–THBS1–SASP axis represents the central signaling pathway that is highlighted in our study; however, it is likely part of a broader regulatory network.

Thrombospondin-1 (THBS1) is a large, multifunctional ECM glycoprotein that plays crucial roles in tissue remodeling and cell–matrix interactions, as well as in the regulation of fibrosis, inflammation, and cellular senescence [34–36,45]. By integrating CUT&Tag and RNA-seq datasets, we identified THBS1 as the top candidate inflammatory regulator. Our data suggest that THBS1 may accelerate NP cell senescence and promote fibrotic degeneration of the intervertebral disc through inflammation-related mechanisms.

Our study has limitations. Firstly, while our findings suggest that mitophagy induces metabolic reprogramming in NP cells, the precise molecular mechanisms remain to be fully elucidated. Secondly, we identified H3K18la as a functionally major histone lactylation site modulated by mitophagy. However, other lactylation sites may also contribute to the transcriptional regulation. Finally, THBS1 was identified as the most likely inflammation-regulating gene downstream of H3K18la, although other potential inflammatory regulatory mechanisms may also exist. Future studies will be needed to further delineate the broader epigenetic regulatory network and clarify whether additional histone lactylation sites or downstream effectors contribute to disc degeneration.

## Conclusion

Collectively, we find that activation of mitophagy by TJ0113 reprograms the metabolic profile of senescent NP cells, reducing lactate production. This decrease leads to diminished enrichment of H3K18la at the THBS1 promoter, suppressing THBS1 expression and consequently interrupting downstream inflammatory cascades during IVDD progression. Our study highlights the critical role of a metabolic–epigenetic cascade in mediating the beneficial effects of mitophagy on IVDD, not only providing novel insights into the molecular mechanisms of disc degeneration but also laying a translational medical foundation on developing mitophagy-targeted therapies.

## Materials and Methods

### Human subjects

Human NP tissues were obtained from patients at the Department of Orthopedics, the Second Affiliated Hospital of Zhejiang University School of Medicine. Donors included individuals diagnosed with lumbar vertebral fracture, idiopathic scoliosis, lumbar disc herniation, or lumbar spondylolisthesis. All participants provided written informed consent permitting the use of discarded tissues for research purposes. NP samples graded as grade I or II were categorized as nondegenerated, sourced from patients with lumbar vertebral fracture or idiopathic scoliosis, whereas samples graded as grade IV were designated as degenerative, collected from patients with lumbar disc herniation or lumbar spondylolisthesis. The characteristic details of volunteers enrolled in this study were provided in Table S1. The study protocol was reviewed and approved by the Ethics Committee of the Second Affiliated Hospital of Zhejiang University School of Medicine (approval number: 2023-0840).

### Isolation of human NP cells

Human NP tissues were minced into small pieces and rinsed twice with PBS. The tissue fragments were digested with 2 mg/ml type II collagenase (17101015, Thermo Fisher) in Dulbecco’s modified Eagle’s medium (DMEM) for 40 min. After digestion, samples were centrifuged at 1,000 rpm for 5 min and washed twice with phosphate-buffered saline (PBS). The isolated human NP cells were cultured in DMEM supplemented with 15% fetal bovine serum (FBS) and 1% penicillin–streptomycin (100 U/ml) under 5% CO₂ at 37 °C. NP cells derived from the nondegenerated group at P2 were used as young controls, while those from the same group at passage 8 represented aged NP cells. NP cells obtained from the degenerated group at P2 were defined as degenerated NP cells.

### Reagents and antibodies

TJ0113 [30,31], a long-acting mitophagy activator, is a structurally optimized derivative of UMI-77 [15], which is currently undergoing phase 2 clinical trials (NCT06596005), and was generously provided by the research group of H. Xia. FX-11 (catalog no. HY-16214), L-Lac (catalog no. HY-Y0479), and THBS1 recombinant protein (catalog no. HY-P70725) were purchased from MedChemExpress. All antibodies utilized in this study are detailed in Table S5.

### Study animals and IVDD model

All animal experiments followed the ethical guidelines of Zhejiang Academy of Medical Sciences and were approved by the Zhejiang Center of Laboratory Animals (ZJCLA) Institutional Animal Care and Use Committee (approval no. ZJCLA-IACUC-20011118). Sprague–Dawley rats were purchased from Zhejiang Academy of Medical Sciences and housed under specific pathogen-free (SPF) conditions at 23 ± 3 °C, 55 ± 10% humidity, with a 12-h light/dark cycle. Food and water were available ad libitum.

Needle puncture-induced IVDD model: The rats (approximately 300 g) were anesthetized by intraperitoneal injection of 1% pentobarbital sodium. After anesthesia, the intervertebral discs (coccygeal vertebrae Co7/8, Co8/9, and Co9/10 in each rat) of the rats were punctured with a 20-gauge sterile needle (Hamilton). The puncture needle penetrated the annulus fibrosus on one side of the intervertebral disc into the NP region, rotated 360° and held for 30 s to induce degeneration.

Rats were randomly assigned to 4 groups, and treatment commenced 2 weeks after needle puncture: Sham group (no treatment), Degeneration group (20-gauge puncture + 2 μl of PBS weekly intradiscal injection), TJ0113 (10 mg/ml, 20 mg/ml) group (20-gauge puncture + 2 μl of TJ0113 weekly intradiscal injection), and UA (5 mg/ml, 7.5 mg/ml) group (20-gauge puncture + 2 μl of UA weekly intradiscal injection). Rat tissue samples were collected at 4 and 12 weeks.

Rats were randomly assigned to 4 groups, and treatment was initiated 2 weeks after puncture following a cyclic 4-week administration regimen: Sham group (no treatment), Degeneration group (20-gauge puncture + 2 μl of PBS weekly intradiscal injection), TJ0113 (10 mg/ml) group (20-gauge puncture + 2 μl of PBS intradiscal injection in week 1 + 2 μl of TJ0113 intradiscal injection weekly in weeks 2 to 4), and TJ0113 (10 mg/ml) + L-Lac (5 mM) group (20-gauge puncture + 2 μl of L-Lac intradiscal injection in week 1 + 2 μl of TJ0113 intradiscal injection weekly in weeks 2 to 4). We used a 5-μl Hamilton microliter syringe to perform solution delivery. Rat tissue samples were collected at 12 weeks (after 3 complete cycles).

### Quantitative reverse transcription PCR

Total RNA was isolated using TRIzol (Thermo Fisher), and concentration was measured by NanoDrop 2000. cDNA was synthesized from ≤1 μg of RNA using the Double-Strand cDNA Synthesis Kit (Takara). qPCR was performed with SYBR Green Master Mix (Takara) on a StepOnePlus System, with samples running in triplicate. Expression levels were normalized to 18*S* and calculated by the 2−ΔΔCt method. Primers were synthesized by Sangon Biotech (Table S2). Cycling conditions: 95 °C for 30 s, then 40 cycles of 95 °C for 5 s and 60 °C for 30 s.

### RNA-seq and analysis

The RNA-seq experiments and data analysis in this study were performed by Hangzhou Lianchuan Biotechnology Co. Ltd. The specific experimental procedures and analysis methods were conducted following previously published protocols [46].

### Metabolomics sequencing and data analysis

NP cells were divided into 2 groups: the TJ0113 treatment group and the control group. For the treatment group, NP cells were incubated with TJ0113 for 48 h, while the control group received vehicle treatment. At the end of the treatment, cells were collected, and each biological replicate contained approximately 1 × 10^7^ cells. A total of 5 independent replicates per group were prepared.

Metabolite extraction, metabolomics sequencing, and subsequent data analysis were performed by Metware Biotechnology Co. Ltd. (Wuhan, China) using a widely targeted metabolomics platform based on ultra-performance liquid chromatography coupled with tandem mass spectrometry (UPLC–MS/MS). Raw data were processed for peak detection, alignment, and quantification. Metabolites were annotated against in-house and public databases. Multivariate statistical analyses, including principal components analysis (PCA) and orthogonal partial least squares discriminant analysis (OPLS-DA), were performed to evaluate group differences. Significantly altered metabolites were identified based on a variable importance in projection (VIP) score > 1 and *P* < 0.05 (Student’s *t* test).

### Cleavage under targets and tagmentation

Aged NP cells (100,000) treated with or without TJ0113 (5 μM, 48 h) were collected and then subjected to perform CUT&Tag assay (12598ES12, Yeasen Biotech). Cell lysates were incubated at room temperature with concanavalin A-coated magnetic beads for 10 min and then with the primary antibody against H3K18la (1:100; PTM-1427RM, PTM BIO) for 2 h, with the mix of secondary antibodies and pA/G-Tn5 adapter for 0.5 h. After 30-min incubation with proteinase K, terminate solution, and spike-in mix at 55 °C, DNA was extracted. Libraries were prepared using the TruePrep Index Kit V2 for Illumina (TD202, Vazyme) according to the manufacturer’s protocol. The libraries were pooled and subjected to paired-end 150-base pair sequencing on the Illumina Novaseq 6000 platform. Data visualization was conducted using Integrative Genomics Viewer (IGV).

### Chromatin immunoprecipitation

NP cells were cross-linked with 1% formaldehyde for 10 min, followed by quenching with glycine. Nuclear lysates were sonicated to fragment chromatin. Approximately 100 μg of protein from each lysate was incubated overnight at 4 °C with 5 μl of H3K18la antibody and ChIP-grade magnetic beads. After reversing the cross-links, DNA was purified using the QIAquick PCR Purification kit. qPCR was then performed using specific primers (listed in Table S3) and SYBR Green Mix on a thermocycler.

### siRNA transfection

When cells reached 70% to 80% confluence, the culture medium was refreshed with 1.8 ml of new medium. Then, a transfection mixture containing 150 μl of serum-free DMEM, 5 μl of 20 μM siRNA, and 35 μl each of Reagent A and Reagent B (DN001-10, D-Nano Therapeutics) was added to the medium. SiRNA sequences were documented in Table S4.

### Immunofluorescence

Deparaffinized sections from rat samples were heated in sodium citrate buffer solution (pH 6.5) for 1 h at 70 °C for antigen retrieval and incubated in the sealing solution (5% bovine serum albumin in PBS) for 60 min at room temperature. Blocked sections were then incubated with primary antibodies (listed in Table S5) overnight at 4 °C. After 3 PBS washes, sections were incubated with Alexa Fluor-conjugated secondary antibodies (1:1,000; Thermo Fisher) for 1 h at 37 °C. Following mounting with 4′,6-diamidino-2-phenylindole (DAPI)-containing medium (F6057, Sigma), fluorescence images were acquired using a Leica microscope. Positive staining areas and cell proportions were quantified with ImageJ software.

### JC-1 staining

NP cells from various groups were seeded into 12-well plates. After washing once with PBS, cells were incubated with JC-1 working solution at 37 °C for 20 min, followed by 2 washes with staining buffer. Fluorescence images were then captured using a Leica fluorescent microscope.

### SA-β-gal staining

Aged and degenerated NP cells were cultured in 6-well plates and treated with or without TJ0113 for 48 h. After treatment, cells were rinsed once with PBS and fixed at room temperature for 15 min using a specialized fixative. They were then incubated overnight at 37 °C without CO₂ in the working solution, followed by 2 PBS washes. Images were acquired using a light microscope.

### Western blotting analysis

NP cells were lysed on ice for 30 min in radioimmunoprecipitation assay (RIPA) buffer with protease and phosphatase inhibitors, with intermittent vortexing. Lysates were centrifuged at 14,000 rpm for 10 min at 4 °C, and supernatants were collected. Proteins were mixed with loading buffer, separated by sodium dodecyl sulfate–polyacrylamide gel electrophoresis (SDS-PAGE), and then transferred to polyvinylidene difluoride (PVDF) membranes. Membranes were blocked with 10% skim milk in tris-buffered saline with Tween (TBST) for 1 h, incubated overnight at 4 °C with primary antibodies (Table S5), washed, and incubated with horseradish peroxidase (HRP)-conjugated secondary antibodies for 1 h at room temperature. Detection was performed using enhanced chemiluminescence (ECL) reagents and imaged with a Bio-Rad system.

### Lactate measurement

To assess lactate production, NP cell lysates were analyzed using a lactate detection kit (Abbkine, KTB1100) as instructed by the supplier. Following a 30-min incubation at ambient temperature, absorbance was read at 450 nm using an enzyme-labeled analyzer.

### Measurement of ECAR

ECAR measurements were performed using the Seahorse XFe96 Analyzer (Agilent) following 48-h treatment of aged NP cells with TJ0113 or UA. Cells (30,000 per well) were seeded in XF96 plates and incubated overnight. The next day, the medium was replaced with XF base medium containing 2 mM glutamine, and cells were equilibrated for 1 h in a CO₂-free incubator. ECAR was recorded at baseline and after sequential injections of glucose (10 mM), oligomycin (1 μM), and 2-deoxy-D-glucose (2-DG) (50 mM). Wave software was used for data analysis.

### Measurement of OCR

The Seahorse XFe96 Analyzer (Agilent) was used to evaluate OCR in aged NP cells treated with or without TJ0113 for 48 h. Cells (30,000 per well) were plated in XF96 plates and incubated overnight. Before measurement, the culture medium was replaced with XF assay medium (with 1 mM pyruvate, 2 mM glutamine, 10 mM glucose) and incubated at 37 °C without CO₂ for 1 h. OCR was monitored after successive injections of oligomycin (1.5 μM), carbonyl cyanide 4-(trifluoromethoxy)phenylhydrazone (FCCP) (1 μM), and rotenone/antimycin A (0.5 μM). Data were processed using Wave software.

### Flow cytometry

After seeding NP cells into 6-well plates, TJ0113 was administered or omitted for a 48-h incubation period. Subsequently, cells were collected, washed with PBS, and subjected to apoptosis detection via Annexin V and propidium iodide (PI) staining using apoptosis kit (C1062S, Beyotime).

### Transmission electron microscopy

NP cells were treated with or without TJ0113 and immediately fixed with 2.5 % glutaraldehyde. Subsequently, samples were fixed in 0.1 M phosphate buffer (PB; pH 7.4) containing 1% osmium tetroxide for 2 h at room temperature in the dark. After fixation, they were dehydrated through a graded ethanol series, permeabilized with acetone, and embedded overnight at 37 °C. Polymerization was carried out at 60 °C for 48 h. Resin-embedded blocks were stained with toluidine blue for localization under a light microscope, sectioned into ultrathin slices, and further stained with uranyl acetate and lead citrate before observation using a transmission electron microscope.

### Statistical analysis

Mean ± SD was used to express data. Statistical evaluation was conducted in GraphPad Prism 10.4.0. Experiments were repeated at least 3 times. A 2-tailed Student’s *t* test was used for comparisons between 2 groups, and one-way analysis of variance (ANOVA) with Bonferroni correction was applied for analyses involving 3 or more groups. Statistical significance was defined as *P* < 0.05.

## Data Availability

Relevant data are available from the corresponding author on reasonable request.
