# Supplementary material for: Mitophagy Reprograms Lactate Metabolism to Suppress THBS1 via H3K18la Reduction, Alleviating Intervertebral Disc Degeneration
Source: Research (Wash D C). 2025 Nov 5;8:0957. doi: 10.34133/research.0957 (PMC12586853; doi:10.34133/research.0957)
Supplement: Supplementary 1 — Figs. S1 to S9 Tables S1 to S5 [file research.0957.f1.zip › Supplemental Figures and Tables.docx]

**Figure S1**

**
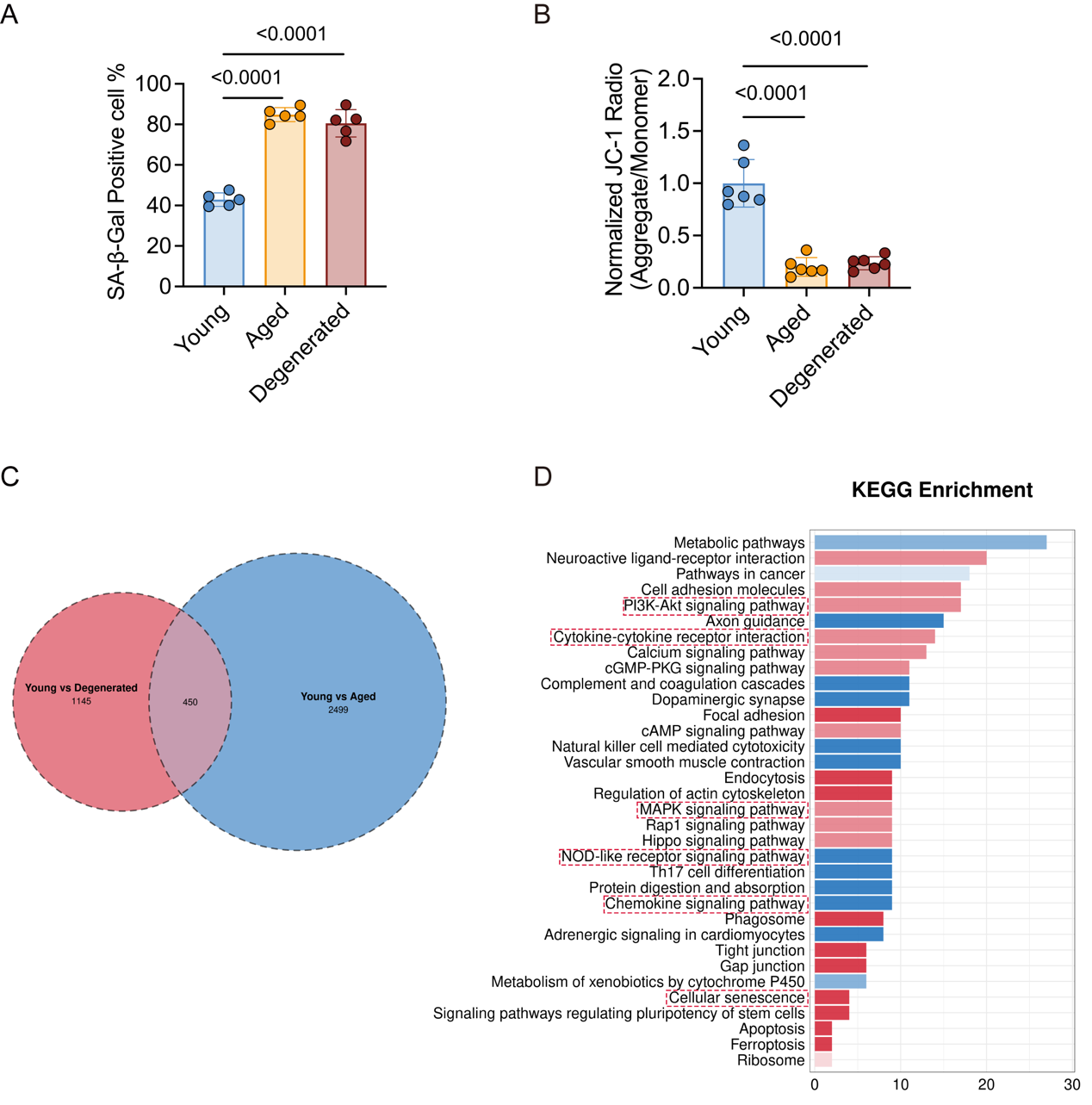
**

**Figure S1.** (A) Quantitation of β-gal staining. (B) Quantitation of JC-1staining. (C) The overlapping genes between the two RNA-seq datasets were illustrated by a Venn diagram. (D) KEGG enrichment analysis of the overlapping genes. At least 3 independent experiments were performed. Data are expressed as mean ± SD. Statistical significance is denoted in the graphs.

**Figure S2**

**
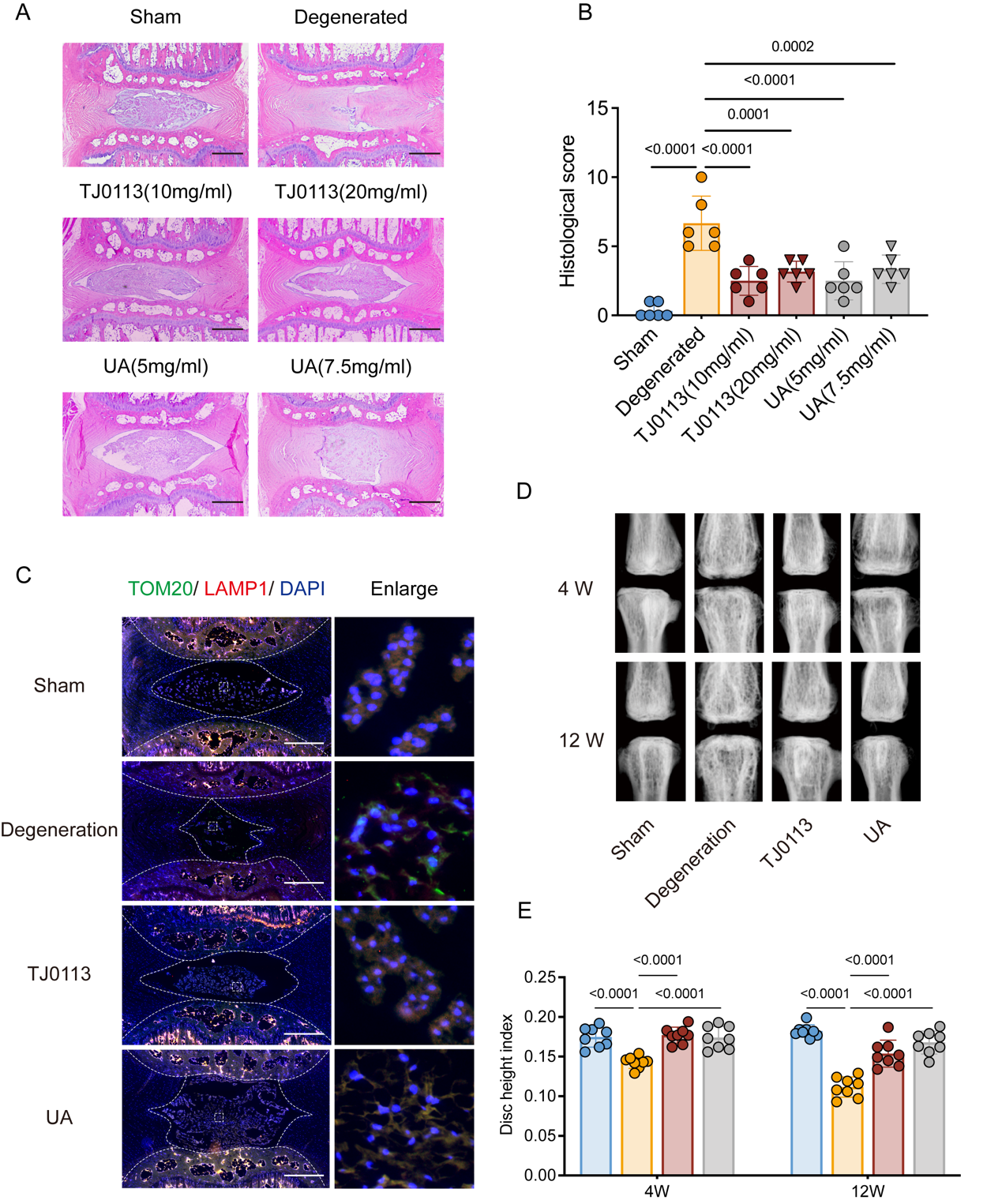
**

**Figure S2.** (A-B) Representative images and quantitation of H&E staining of rat coccygeal IVDs at 4 weeks after TJ0113 (10mg/ml; 20mg/ml) and UA (5mg/ml; 7.5mg/ml) treatment. Scale bar: 1 mm. (C) Representative immunofluorescence images of Tom20 and Lamp1 colocalization in rat coccygeal IVDs at 4 weeks after treatment. Scale bar: 500 μm. (D) Representative x-ray images of rat coccygeal IVDs of different groups. (E) Disc height index (n=8) of rat coccygeal IVDs. Data are expressed as mean ± SD. Statistical significance is denoted in the graphs.

**Figure S3**

**Figure S3.** Flow cytometric analysis of aged NP cells treated with different concentrations of TJ0113. At least 3 independent experiments were performed. Data are expressed as mean ± SD. Statistical significance is denoted in the graphs.

**Figure S4**

**
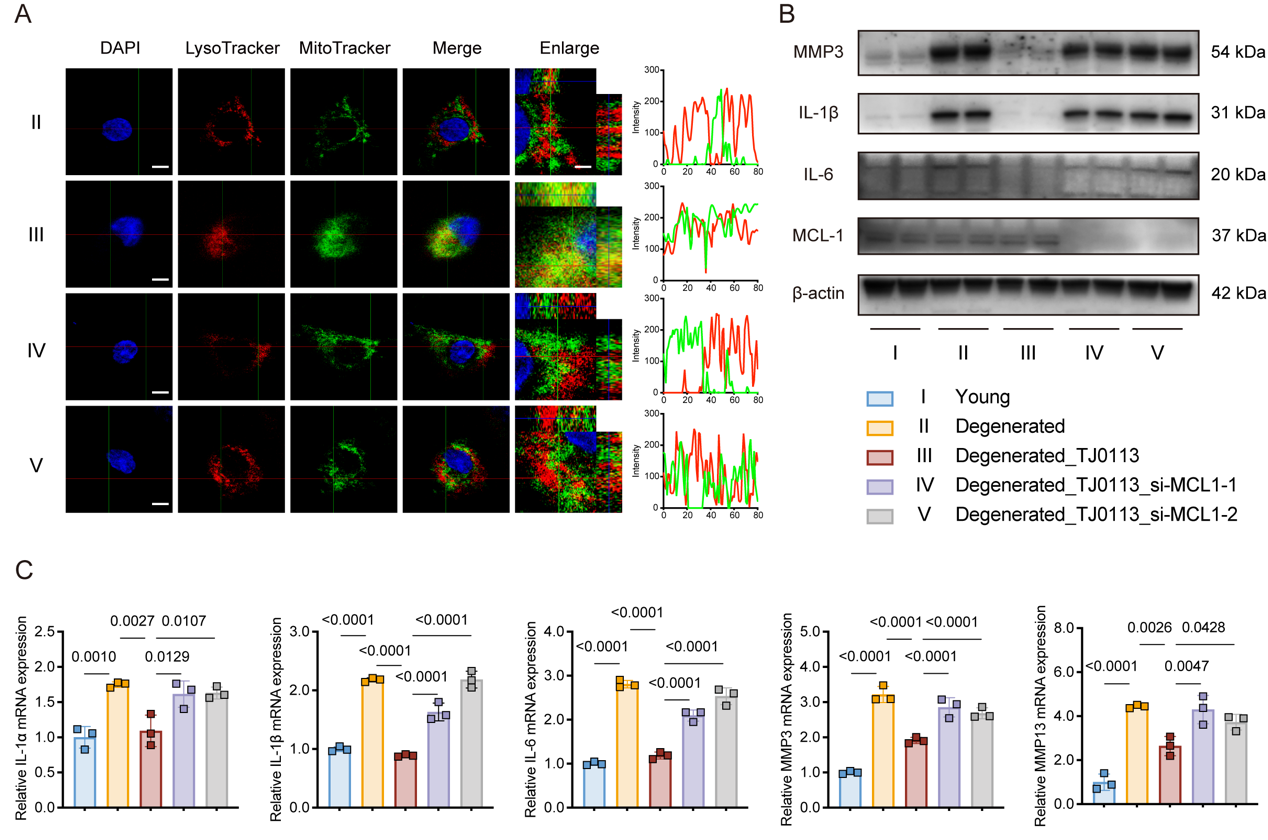
**

**Figure S4.** (A) Immunofluorescence analysis and quantification of mitotracker and lysotracker in degenerated NP cells treated with or without TJ0113 (5 μM) after knocking down MCL1. Scale bars, 10 μm. (B) Western blot analysis of SASP factors (IL-1β, IL-6, MMP3) and MCL1 in young and degenerated NP cells treated with or without TJ0113 (5 μM, 48 hours) after knocking down MCL1. β-actin served as loading control. (C) qRT-PCR analysis of SASP factors (IL-1α, IL-1β, IL-6, MMP3, MMP13) in young and degenerated NP cells treated with or without TJ0113 (5 μM, 48 hours; n=3) after knocking down MCL1.At least 3 independent experiments were performed. Data are expressed as mean ± SD. Statistical significance is denoted in the graphs.

**Figure S5**


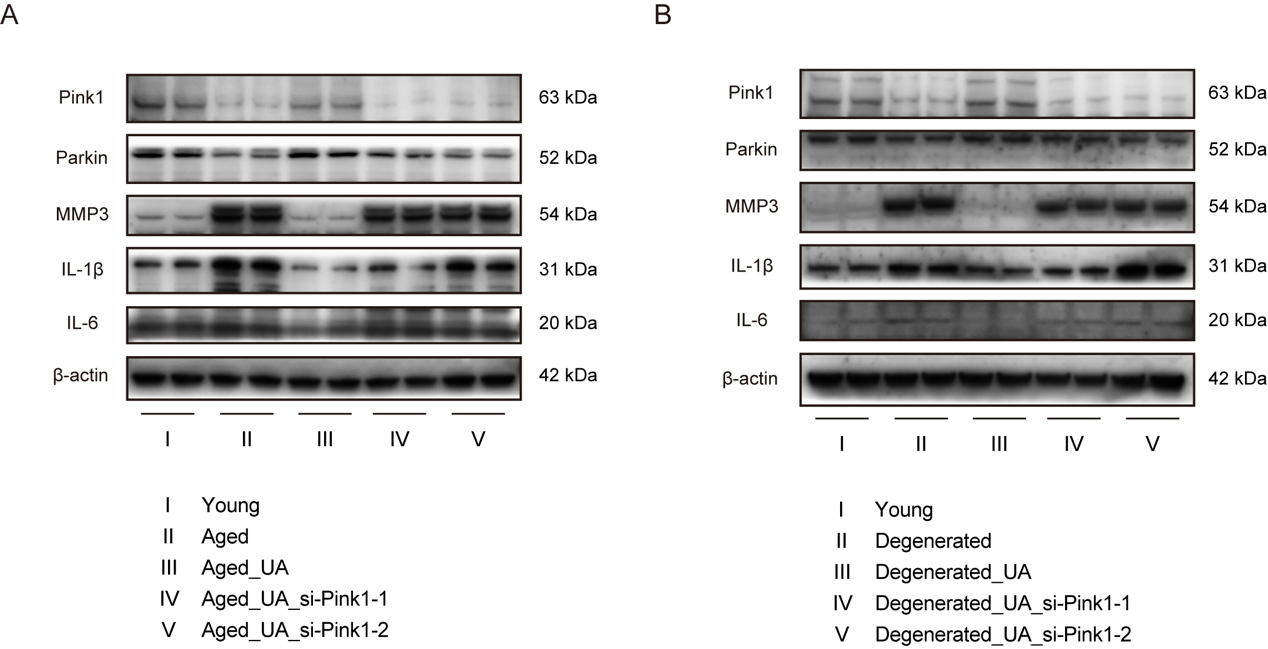


**Figure S5.** (A) Western blot analysis of SASP factors (IL-1β, IL-6, MMP3), Pink1 and Parkin in young and aged NP cells treated with or without TJ0113 (5 μM, 48 hours) after knocking down Pink1. β-actin served as loading control. (B) Western blot analysis of SASP factors (IL-1β, IL-6, MMP3), Pink1 and Parkin in young and degenerated NP cells treated with or without TJ0113 (5 μM, 48 hours) after knocking down Pink1. β-actin served as loading control. At least 3 independent experiments were performed. Data are expressed as mean ± SD. Statistical significance is denoted in the graphs.

**Figure S6**


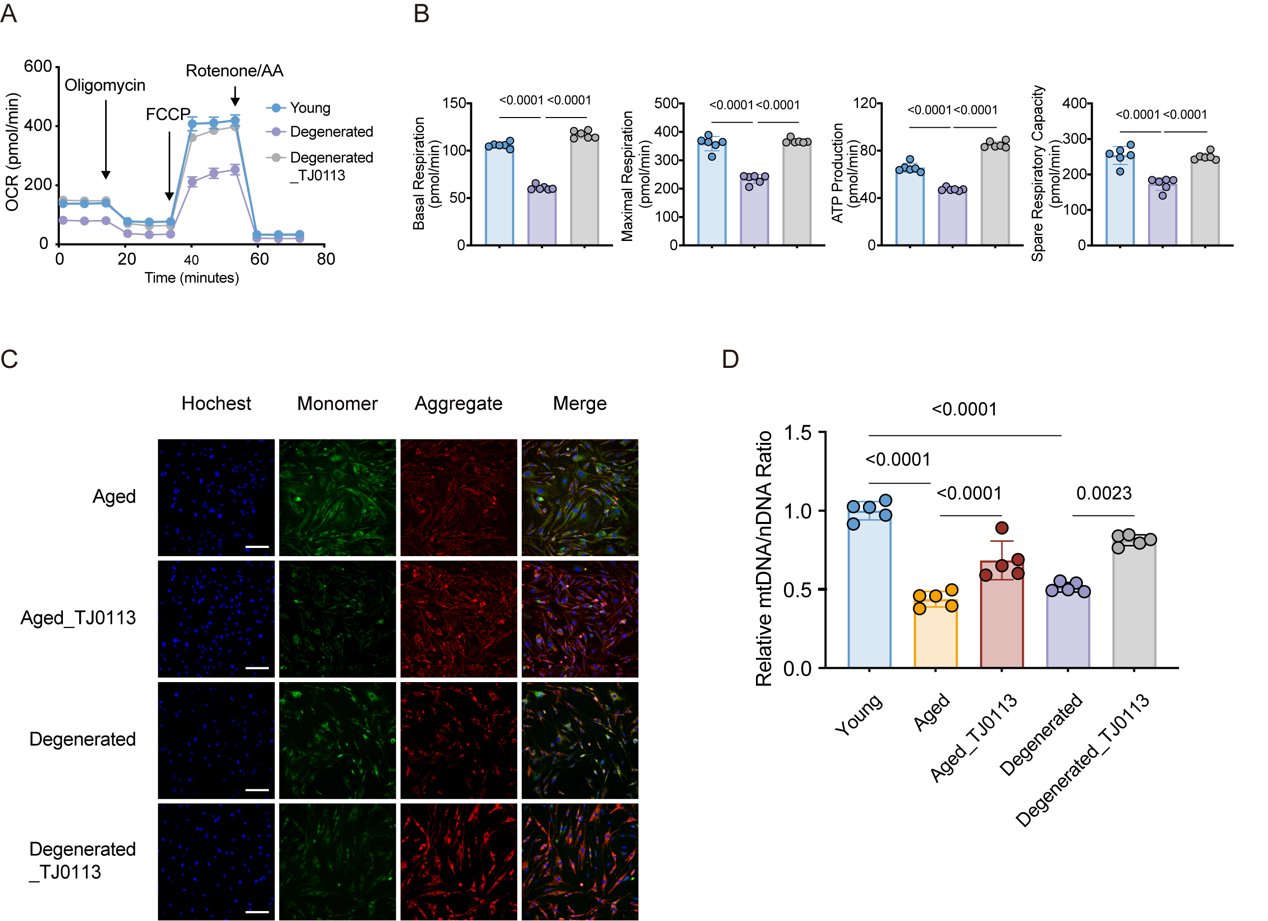


**Figure S6.** (A-B) Seahorse metabolic analysis (OCR) of degenerated NP cells treated with TJ0113 or not (5 μM, 48 h). Young group served as control. (C) Representative images of JC-1 staining of aged and degenerated NP cells treated with or without TJ0113 (5 μM, 48 h). Scale bar: 50 μm. (D) mtDNA levels and nDNA levels in NP cells form different groups were measured by qRT-PCR following TJ0113 treatment (5 μM, 48 h; n=5). At least 3 independent experiments were performed. Data are expressed as mean ± SD. Statistical significance is denoted in the graphs.

**Figure S7**


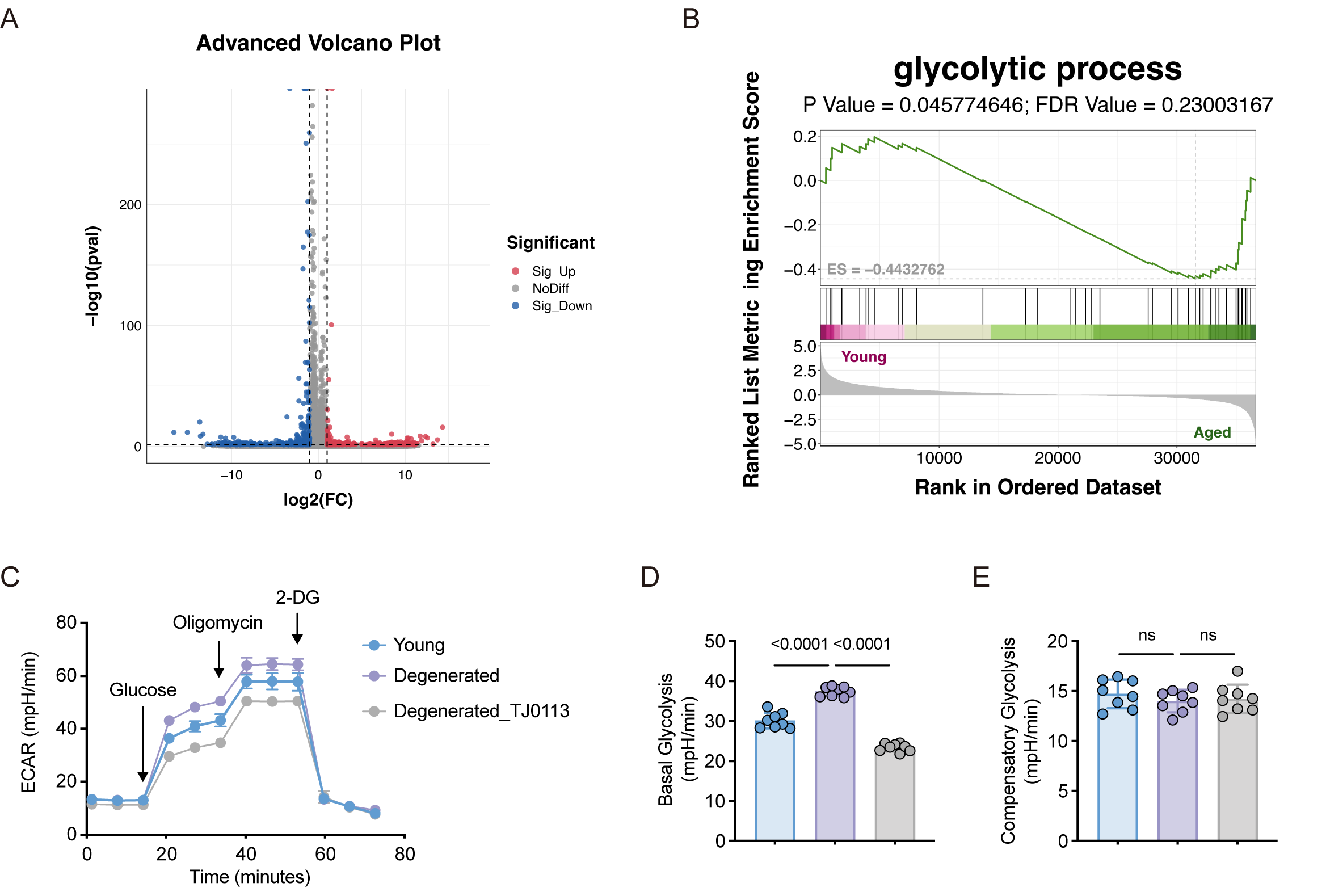


**Figure S7.** (A) ﻿Volcano plot of all transcripts between the aged_TJ0113 group and the aged group. (B) GSEA revealed significant enrichment of the “glycolytic process” in aged group. (C-E) Seahorse metabolic analysis (ECAR) of degenerated NP cells treated with TJ0113 or not (5 μM, 48 h). Young NP cells served as control. At least 3 independent experiments were performed. Data are expressed as mean ± SD. Statistical significance is denoted in the graphs.

**Figure S8**


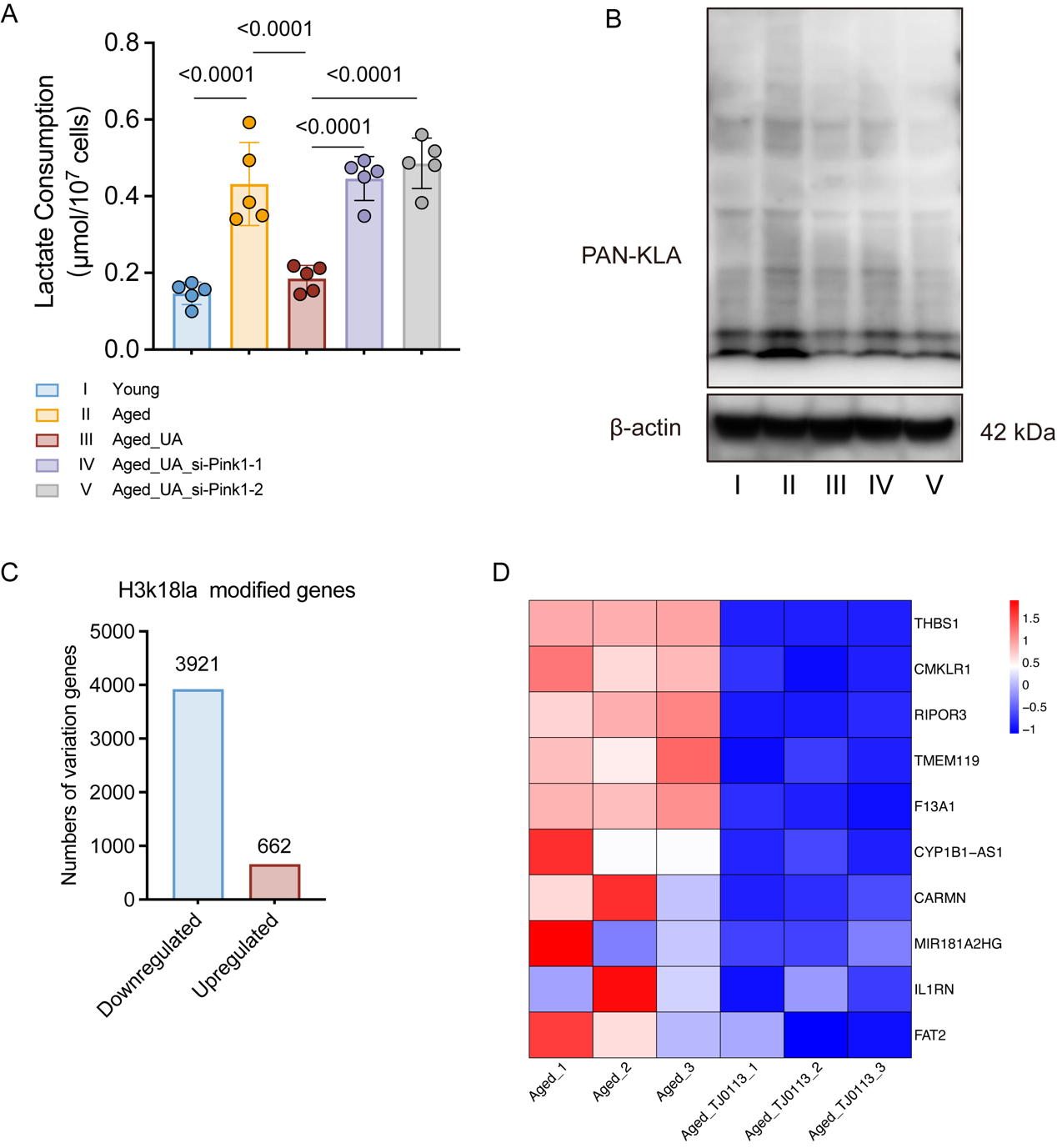


**Figure S8.** (A) Lactate levels of the young and aged NP cells treated with or without UA (10 μM, 48 h; n=5) after knocking down Pink1. (B) Western blot analysis of Pan-Kla in aged NP cells treated with or without UA (10 μM, 48 h) after knocking down Pink1. β-actin served as loading controls. (C) H3K18la modified genes that are differently regulated in aged NP cells treated with or without TJ0113 (5 μM, 48 h). (D) Heatmap of promoter-associated H3K18la-modified genes, ordered by Q-values of RNA-seq. At least 3 independent experiments were performed. Data are expressed as mean ± SD. Statistical significance is denoted in the graphs.

**Figure S9**


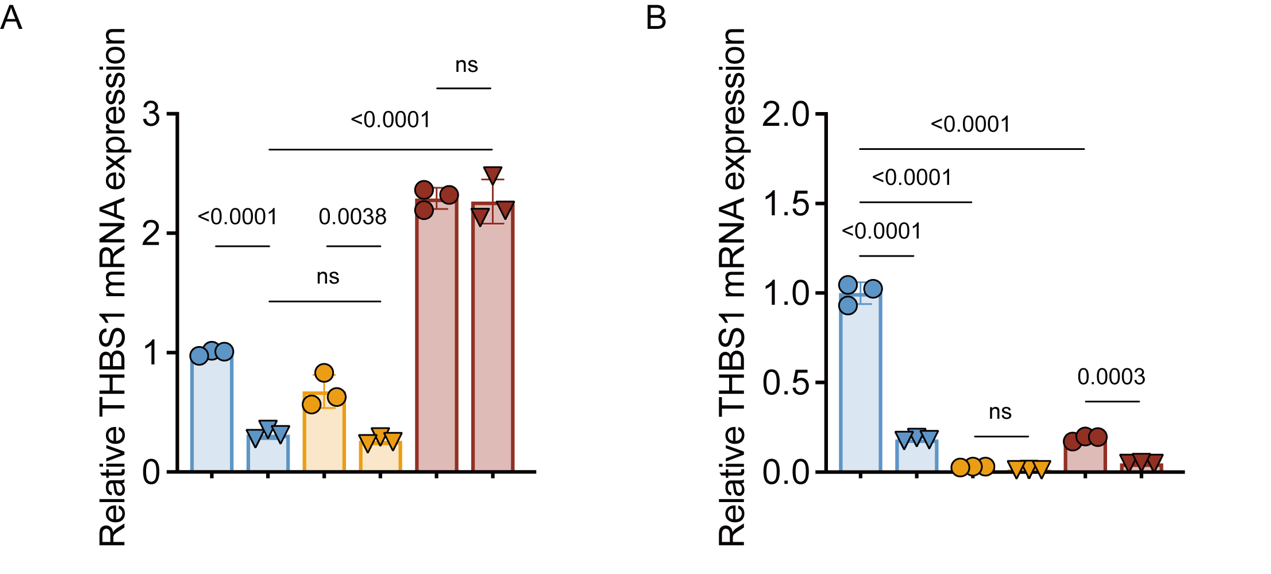


**Figure S9.** (A) qRT-PCR analysis of THBS1 in aged NP cells with treatment as Figure 6B-C. (B) qRT-PCR analysis of THBS1 in control and THBS1 knockdown aged NP cells treated with or without TJ0113 (5 μM, 48 h) (n=3). At least 3 independent experiments were performed. Data are expressed as mean ± SD. Statistical significance is denoted in the graphs.

**Supplemental Tables**

**TableS1 Demographic data of patients.**

| **Patients No.** | **Gender** | **Age** | **Pfirrmann MRI grade** | **Levels** |
| --- | --- | --- | --- | --- |
| **1** | **Male** | **65** | **IV** | **L4-L5** |
| **2** | **Male** | **59** | **IV** | **L4-L5** |
| **3** | **Male** | **71** | **IV** | **L4-L5** |
| **4** | **Female** | **62** | **IV** | **L5-S1** |
| **5** | **Male** | **53** | **IV** | **L3-L4** |
| **6** | **Female** | **51** | **IV** | **L4-L5** |
| **7** | **Female** | **68** | **IV** | **L4-L5** |
| **8** | **Male** | **69** | **IV** | **L5-S1** |
| **9** | **Male** | **50** | **IV** | **L4-L5** |
| **10** | **Female** | **53** | **IV** | **L4-L5** |
| **11** | **Female** | **46** | **IV** | **L5-S1** |
| **12** | **Male** | **55** | **IV** | **L4-L5** |
| **13** | **Female** | **59** | **IV** | **L5-S1** |
| **14** | **Male** | **65** | **IV** | **L5-S1** |
| **15** | **Male** | **53** | **IV** | **L4-L5** |
| **16** | **Male** | **15** | **I** | **T10-T11** |
| **17** | **Male** | **21** | **I** | **T10-T11** |
| **18** | **Female** | **28** | **II** | **L5-S1** |
| **19** | **Male** | **16** | **I** | **L1-L2** |
| **20** | **Female** | **25** | **I** | **L2-L3** |
| **21** | **Male** | **19** | **II** | **L2-L3** |
| **22** | **Female** | **15** | **I** | **T12-L1** |
| **23** | **Female** | **17** | **I** | **L1-L2** |
| **24** | **Female** | **32** | **I** | **L3-L4** |

**TableS2 RT-PCR primers used.**

| **Gene** | **Forward (5’ to 3’)** | **Reverse (5’ to 3’)** |
| --- | --- | --- |
| THBS1 | AGACTCCGCATCGCAAAGG | TCACCACGTTGTTGTCAAGGG |
| MMP13 | GCCATTACCAGTCTCCGAGG | TACGGTTGGGAAGTTCTGGC |
| MMP3 | AGTCTTCCAATCCTACTGTTGCT | TCCCCGTCACCTCCAATCC |
| ADAMTS5 | GAACATCGACCAACTCTACTCCG | CAATGCCCACCGAACCATCT |
| IL-6 | CCAGGAGCCCAGCTATGAAC | CCCAGGGAGAAGGCAACTG |
| IL-8 | AAGGAAAACTGGGTGCAGAG | ATTGCATCTGGCAACCCTAC |
| IL-1α | AACCAGTGCTGCTGAAGGA | TTCTTAGTGCCGTGAGTTTCC |
| IL-1β | CTGTCCTGCGTGTTGAAAGA | TTGGGTAATTTTTGGGATCTACA |
| ND1 | ﻿CACCCAAGAACAGGGTTTGT | ﻿TGGCCATGGGTATGTTGTTAA |
| 18S | GCGGCGGAAAATAGCCTTTG | GATCACACGTTCCACCTCATC |

**TableS3 ChIP primer sequences used**

| **Gene** | **Forward (5’ to 3’)** | **Reverse (5’ to 3’)** |
| --- | --- | --- |
| THBS1 | GGGCACCGACTTTTCTGAGA | CATTCCTGGGGATTCCTCCG |

**TableS4 siRNA sequences used in siRNA transfection**

| **Sequence name** | **Sense (5’-3’)** | **Antisense (5’-3’)** |
| --- | --- | --- |
| hTHBS1-1 | GCGUGUUUGACAUCUUUGA | UCAAAGAUGUCAAACACGC |
| hTHBS1-2 | CCCUGUUUGUGCAGGAAGA | UCUUCCUGCACAAACAGGG |
| hPINK1-1 | GGCAGGUUCCUCCAGCGAA | UUCGCUGGAGGAACCUGCC |
| hPINK1-2 | GGACGCUGUUCCUCGUUAU | AUAACGAGGAACAGCGUCC |
| hMCL1-1 | AAACGAAGACGAUGUGAAA | UUUCACAUCGUCUUCGUUU |
| hMCL1-2 | CCAAGAAAGCUGCAUCGAA | UUCGAUGCAGCUUUCUUGG |

**Table S5 Antibodies used**

| **Antibody** | **Company** | **Catalog#** | **Use** |
| --- | --- | --- | --- |
| Rabbit Anti-IL-6 Polyclonal | Proteintech | 21865-1-AP | WB |
| Rabbit Anti-IL-1β Polyclonal | Proteintech | 16806-1-AP | WB |
| Rabbit Anti-MCL1 Polyclonal | Proteintech | 16225-1-AP | WB |
| Rabbit Anti-PINK1 Polyclonal | Proteintech | 23274-1-AP | WB |
| Rabbit Anti- PARK2/Parkin Polyclonal | Proteintech | 14060-1-AP | WB |
| Rabbit Anti-MMP13 Polyclonal | Proteintech | 18165-1-AP | WB |
| Rabbit Anti-P16-INK4A Polyclonal | Proteintech | 10883-1-AP | WB |
| Rabbit Anti-Tom20 Polyclonal | Proteintech | 11802-1-AP | WB |
| Rabbit Anti-Tim23 Polyclonal | Proteintech | 11123-1-AP | WB |
| Rabbit Anti-OPA1 Polyclonal | Proteintech | 27733-1-AP | WB |
| Rabbit Anti-MFN1 Polyclonal | Proteintech | 13798-1-AP | WB |
| Rabbit Anti-MFN2 Polyclonal | Proteintech | 12186-1-AP | WB |
| OXPHOS Cocktail | Proteintech | PK30006 | WB |
| Rabbit Anti-P21 monoclonal | Abcam | ab109199 | WB |
| Rabbit Anti-L-Lactyl Lysine Polyclonal | PTM BIO | PTM-1401RM | WB/IF |
| Rabbit Anti-L-Lactyl-Histone H3 (Lys9) monoclonal | PTM BIO | PTM-1419RM | WB |
| Rabbit Anti-L-Lactyl-Histone H3 (Lys14) monoclonal | PTM BIO | PTM-1414RM | WB |
| Rabbit Anti-L-Lactyl-Histone H3 (Lys18) monoclonal | PTM BIO | PTM-1406RM | IHC-P |
| Rabbit Anti-L-Lactyl-Histone H3 (Lys18) monoclonal | PTM BIO | PTM-1427RM | WB/Cut Tag |
| Rabbit Anti-Histone H3 Polyclonal | Proteintech | 17168-1-AP | WB |
| Rabbit Anti-Histone H4 Polyclonal | Proteintech | 16047-1-AP | WB |
| Rabbit Anti-THBS1 monoclonal | HUABIO | HA721916 | WB/IF |
| Rabbit Anti-MMP3 monoclonal | HUABIO | ET1705-98 | WB |
| Mouse Anti-Beta Actin monoclonal | Proteintech | 66009-1-Ig | WB |
| Mouse Anti-Fis1 monoclonal | Santa Cruz Biotechnology | sc-376446 | WB |
| Mouse Anti-Vinculin monoclonal | Proteintech | 66305-1-Ig | WB |
| Donkey anti-Rabbit IgG (H+L) Highly Cross-Adsorbed Secondary Antibody, Alexa Fluor™ Plus 488 | Invitrogen | A32790 | IF |
| Donkey anti-Mouse IgG (H+L) Highly Cross-Adsorbed Secondary Antibody, Alexa Fluor™ Plus 555 | Invitrogen | A32773 | IF |
| HRP Conjugated AffiniPure Goat Anti-rabbit/mouse IgG (H+L) | Boster | BA1056 | WB |
