# Supplementary figures and images for: Mitophagy Reprograms Lactate Metabolism to Suppress THBS1 via H3K18la Reduction, Alleviating Intervertebral Disc Degeneration
Source: Research (Wash D C). 2025 Nov 5;8:0957. doi: 10.34133/research.0957 (PMC12586853; doi:10.34133/research.0957)

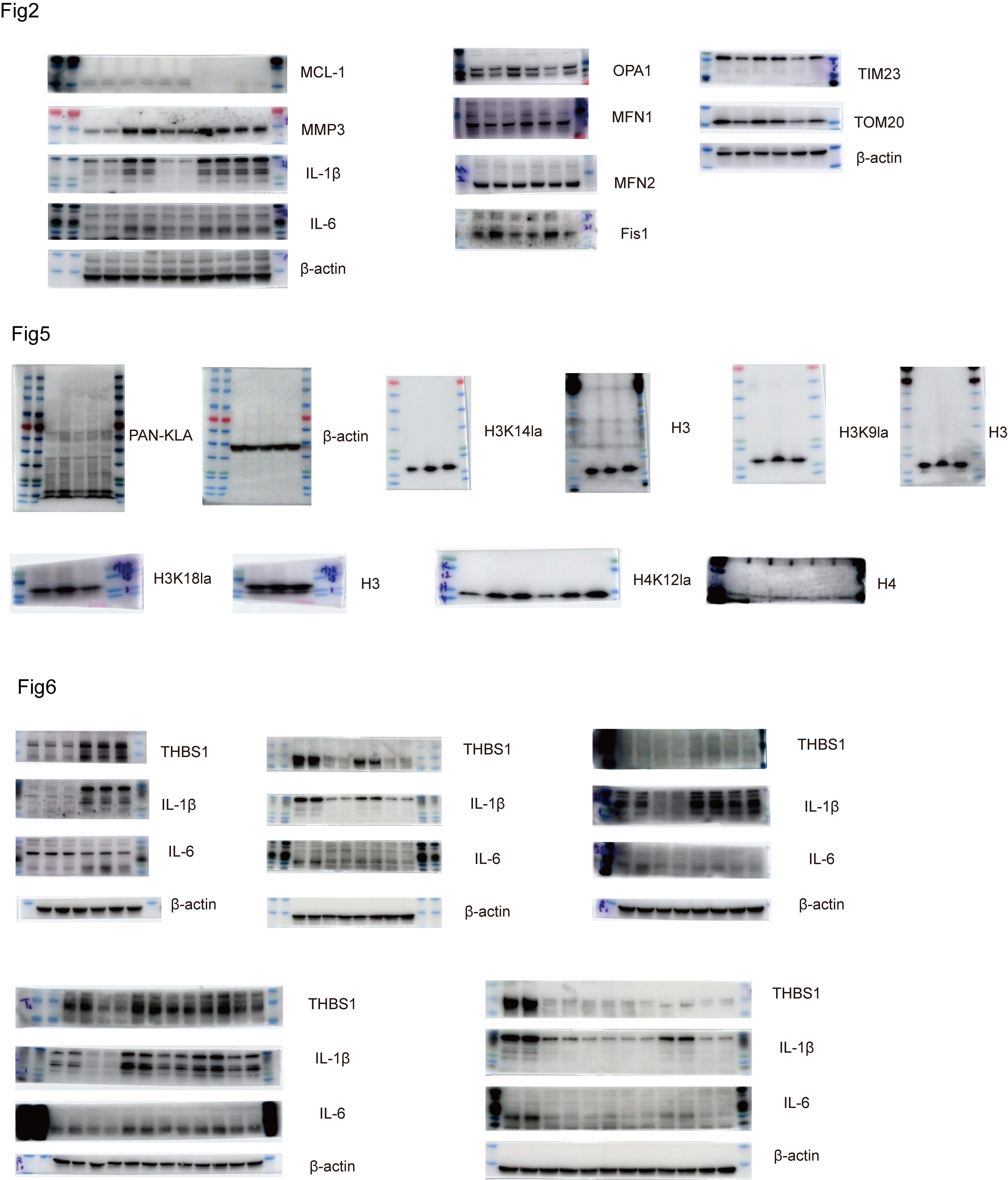

Supplement: Supplementary 1 — Figs. S1 to S9 Tables S1 to S5 [file research.0957.f1.zip › SFig-WB.tif]
